# Post-polymerisation modification of polyolefins through C–H bond activation by frustrated radical pairs

**DOI:** 10.1039/d6cc01531j

**Published:** 2026-07-15

**Authors:** Maartje Otten, Jeroen Hendriks, Léon Witteman, Arnaud Thevenon, Pieter C. A. Bruijnincx

**Affiliations:** a Organic Chemistry & Catalysis, Institute for Sustainable and Circular Chemistry, Faculty of Science, Utrecht University, Universiteitsweg 99 3584 CG Utrecht The Netherlands a.a.thevenon-kozub@uu.nl p.c.a.bruijnincx@uu.nl

## Abstract

Polyethylene and polypropylene are oxyfunctionalised using disilazide/*N*-oxoammonium frustrated radical pair chemistry. C–H activation followed by one-pot *m*CPBA oxidation yields ketone and aldehyde-decorated polymers without backbone cleavage or crosslinking.

## Abstract 2

A polyolefin oxyfunctionalisation method has been developed using frustrated radical pair (FRP) chemistry to introduce ketones and aldehydes into the backbone of polyethylene and polypropylene.

## Graphical abstract

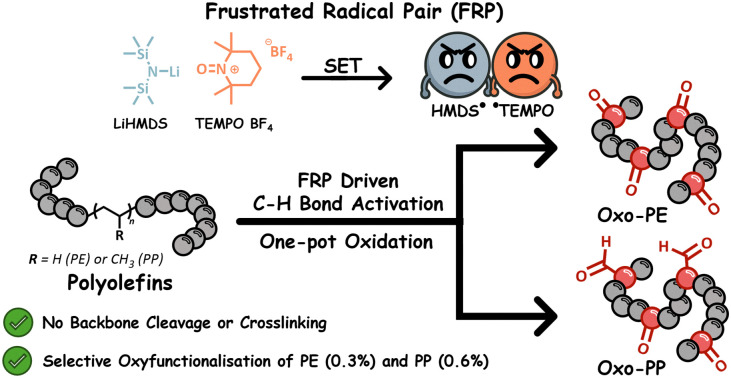

From smart packaging to high-performance composites, modern applications increasingly require materials that combine the robustness of polyolefins with the reactivity and tunability of polar polymers.^1,2^ Thus, oxyfunctionalised polyolefins are increasingly sought after to, for example, expand functional property space or improve end-of-life degradability.^3^ Small amounts of polar functional groups significantly enhance material properties (*e.g.* adhesion), without sacrificing the polyolefin's mechanical ones. While some bottom-up synthesis strategies for oxyfunctionalised polyolefins exist, controlling the degree, position and nature of functionalisation remains a major challenges.^4–10^

Alternatively, oxyfunctional groups can be installed by post-polymerisation modification (PPM), which is arguably more suited for precise lower degrees of (precise) functionalization. Hartwig *et al.* showed small amounts of ketones can be installed on polyethylene (PE) in a two-step PPM precious metal-based approach.^11,12^ Metal-free PPM approaches have also been reported, with high ketone selectivities having been achieved by benzaldehyde-mediated oxidation and nitroarene-mediated photooxidation.^13,14^ Recently, we as well as Taton and Landais separately reported on a photochemical approach to install oxime, nitro or ketone functional groups onto PE without backbone cleavage or crosslinking.^15,16^ LaPointe, Coates *et al.* provided a further elegant example of TEMPO-mediated PE ketonisation *via* radical chemistry,^17^ further highlighting the power of this approach to install oxyfunctional groups on PE (Scheme 1A). However, free-radical systems that operate at lower temperatures and with shorter reaction times remain scarce. Expanding the scope of (radical-based) oxyfunctionalisation to polypropylene (PP) merits attention as such examples remain limited. In PP, C–H activation presents additional regioselectivity challenges, as site-selective radical formation is expected to influence the balance between oxyfunctionalisation and chain degradation. The choice of the C–H activation agent offers the potential to control the regioselectivity. Building on the work of Lin *et al.*, we now translate the use of frustrated radical pairs (FRPs), which have emerged as synthetic tool for C–H bond activation/oxidation in small aliphatic molecules (Scheme 1B) to both chemoselective PE and isotactic PP.^18,19^ Here, we use the hexamethyldisilazide anion (HMDS^−^) and 2,2,6,6-tetramethyl-1-oxo-piperidinium (TEMPO^+^) FRP couple to obtain aminoxylated PE and isotactic PP as intermediate, which can further converted to aldehyde and ketone-functionalized polyolefins using an overall one-pot, robust and metal-free manner (Scheme 1C) without undesired chain cleavage or crosslinking, and with retention of thermal stability. The lack of chain scission upon PP functionalisation is associated with the bulky nature of the HMDS radical leading to regioselective, non-tertiary C–H activation.

**Scheme 1:**
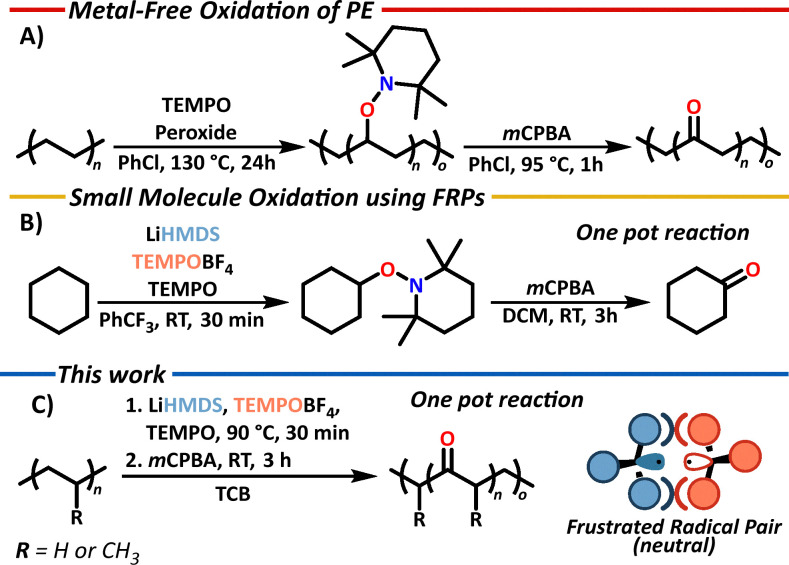
(A) Metal-free PPM example for PE oxidation.^17^ (B) Example of C–H bond activation of cyclohexane using frustrated radical pair (FRP) chemistry.^18^ (C) This work on the metal-free oxidation of polyolefins using FRPs for C–H bond activation and subsequent conversion of the aminoxylated product into a carbonyl with *m*CPBA.

Using the procedure of Lin *et al.*, we first oxidised several PE model compounds to create a spectroscopic dataset to later aid analysis of the oxyfunctionalised PE materials (Scheme 2).^18^ We thus investigated FRP-mediated functionalisation on *n*-undecane and *n*-octadecane, next to cyclohexane, using LiHMDS/TEMPO BF_4_ (with some additional TEMPO), followed by *m*CPBA addition to the same pot. With PE and PP functionalisation in mind, we used 1,2,4-trichlorobenzene (TCB; suitable for polyolefin dissolution) as solvent and ran the model compound oxidations at ambient temperature.

**Scheme 2:**
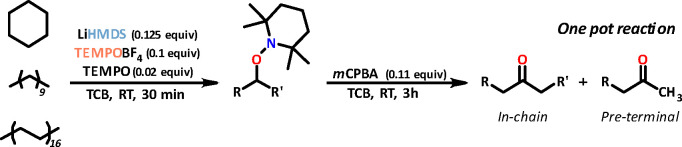
Functionalisation conditions for the FRP oxidation of PE model compounds.

Maximum conversion/yield is based on FRP amount as the substrate was typically used in a ten-fold excess. ^1^H NMR indeed showed the formation of a ketone functional group on the in-chain carbon positions (^1^H NMR: 2.43 ppm for the α-protons), as well as at the pre-terminal carbon position (singlet at 2.15 ppm). The multiplet at ∼1.60 ppm, assigned to β-CH_2_ protons next to a ketone, also proved diagnostic (see below). FTIR further supported ketonisation C=O stretch at 1721 cm^−1^). As expected, longer aliphatic chains gave more in-chain ketones, with in-chain *vs*. preterminal ratios of 1.8 : 1.0 for *n*-undecane and 3.5 : 1.0 for *n*-octadecane. No overoxidation, *e.g. via* Baeyer–Villiger reaction, nor terminal carbon functionalisation to the aldehyde was observed.^20–26^ While yields for cyclohexane and *n*-undecane oxyfunctionalisation (64% and 55%, respectively) were, close to reported yields, only 19% yield was seen for *n*-octadecane, which is tentatively attributed to the observed increased solution viscosity when this higher molecular weight substrate was used.^18^

Having demonstrated the feasibility of C–H bond activation/oxidation on the linear model compounds, we used this approach on self-synthesised low *M*_W_ PE (PE, *M*_w_ = 1.9 kDa and *Đ* = 1.06) as a well-defined polymer sample to easily identify any backbone cleavage or crosslinking events.^15,27^ Oxyfunctionalisation was performed under conditions similar to the model compounds, but at a reaction temperature of 90 °C, necessary to dissolve polyethylene. Oxyfunctionalisation resulted in the isolation of white/beige solids in 86–88% recovered yields. ^1^H NMR analysis showed the same diagnostic ketone signals also observed for the model compounds, demonstrating installation of both in-chain and the pre-terminal ketones (Fig. 2A); we observe a well-defined triplet at 2.41 ppm, which suggests that the ketones are well-spaced on the polymer backbone, as overlapping triplets would be indicative of the opposite. Next to that, no ketones on adjacent carbon atoms are formed as a 2.70 ppm triplet indicative for 1,2-diketones was not observed.^28,29^ Expectedly, functionalisation preferentially occurs on the in-chain position; overall, a functionalisation degree (FG_Ketone_%, *i.e.* per 100 –(CH_2_)_2_–) of 0.33% was found (Table S1, entry 1). Again, a C=O vibration at 1720 cm^−1^ further supports ketone formation on the PE backbone (Fig. 1B). Furthermore, the ^1^H NMR spectra showed no evidence of side reactions such as crosslinking, backbone cleavage or elimination reactions (Fig. S16).

**Fig. 1:**
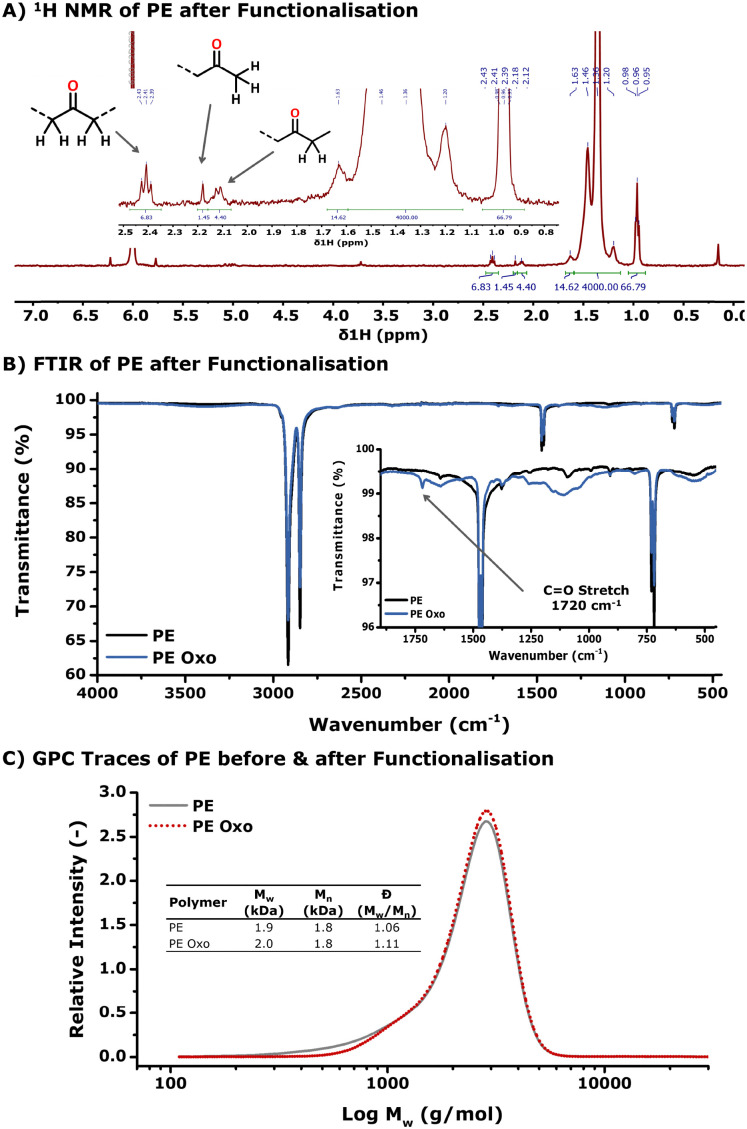
(A) ^1^H NMR spectrum of the product obtained after the oxidation reaction of PE, measured in 1,1,2,2-tetrachloroethane-d_2_ at 120 °C, (B) FTIR spectra of PE and the product obtained after the oxidation reaction of PE and (C) Gel Permeation Chromatography (GPC) traces of PE before and after oxyfunctionalisation, with the corresponding *M*_w_, *M*_n_ and *Đ*.

Gel permeation chromatography (GPC) analysis of the PE Oxo and parent PE materials showed, in line with the NMR spectra, no evidence of significant undesired radical backbone cleavage or crosslinking (Fig. 1C), within the detection limits of the method. There is also no indication for the formation of small alkane fractions (defined as those with an *M*_W_ up to 100 g mol^−1^), as no small molecules were observed by ^1^H NMR in the collected supernatant after filtration. The FRP C–H bond activation approach with the subsequent oxidation is thus well behaved and extends the available radical type strategies able to directly install ketone and ester functional groups without radical-induced backbone cleavage or crosslinking.

We subsequently studied the individual steps and probed what might be limiting the degree of functionalisation. Running the reaction without LiHMDS but otherwise identical to entry 1, did not result in any oxyfunctionalisation (Table S1, entry 8), indicating that HAT from the polymer to the HMDS radical is not taking place *via* any other reagent added and requires the FRP. Similarly, if only *m*CPBA is used also no oxyfunctionalisation is observed (Table S1, entry 9), indicating that C–H activation is required prior to further oxidation. Next to that, the polymer is also not oxyfunctionalised without the oxidation step after the C–H bond activation (Table S1, entry 10).^17^ Last, we explored whether the addition of 12-crown-4 ether during the C–H activation step would increase the functionalisation degree, expecting this crown ether to cause deaggregation of LiHMDS, which typically enhances its solubility and activity.^30,31^ While LiHMDS solubility indeed increased significantly upon the 12-crown-4 addition, no functionalisation of the polymer was observed; the C–H bonds of the crown ether itself most likely get activated instead (Table S1, entry 11). Finally, we attempted to, but could not isolate the intermediate aminoxylated species, previously isolated by LaPointe, Coates *et al.*; this is potentially due to the workup required here to remove the LiOH and HMDS.^17^

Surprisingly, both a lower and higher FRP loadings resulted in lower FG%'s (Table S1, entries 2–4). At lower reaction temperature for the C–H activation step, only trace amounts of functionalisation was observed. We also noted that longer reaction times for either step (Table S1, entries 5 & 6) were not beneficial. Also, increasing the reaction temperature of the second oxidation step to have the polymer fully in solution does not result in any detectable oxyfunctionalisation, likely as a result of olefin formation by elimination rather than oxidation, supported by the resonance at 5.5 ppm (Table S1, entry 7).^17^ Initial variable-temperature NMR studies of the TEMPO BF_4_/HMDS system, conducted in the presence and absence of polyethylene under the applied reaction conditions, reveal the rapid formation of multiple HMDS-derived side products whose formation is accelerated at elevated temperature (Fig. S20–S23). This suggests that competing decomposition pathways consume a significant fraction of the reactive intermediates, preventing higher degrees of functionalization under the applied reaction conditions.^32^

Having demonstrated PE functionalisation, we then switched to isotactic polypropylene (PP, *M*_w_ = 6.3 kDa and *Đ* = 2.37), which offers a mixture of primary, secondary and tertiary carbons for functionalisation (Scheme 3). PP is typically more difficult to functionalise, given that radical-mediated abstraction occurs preferentially at the tertiary C–H position, forming radicals that are prone to β-scission under sufficiently forcing conditions.^33–35^ We hypothesise that the steric bulk of the HMDS-derived radical may disfavour abstraction at this less accessible site, promoting functionalization elsewhere along the polymer backbone and thereby reducing degradation.^36^

**Scheme 3:**
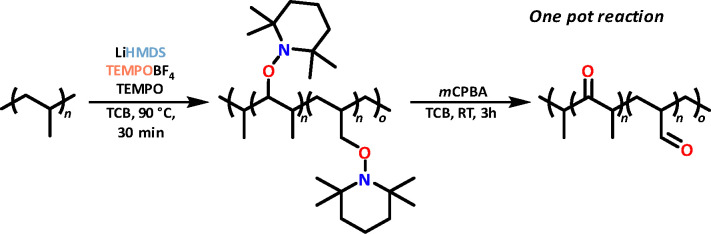
Reaction conditions used for the oxyfunctionalisation of PP using the same FRPs and *m*CPBA approach used for PE.

Using the same protocol, PP was functionalised and isolated as white solid, in similar yield (88%) as for PE. ^1^H NMR analysis showed a doublet at 9.58–9.65 ppm, assigned as an aldehyde on the primary carbon (coupling with the tertiary C–H, Fig. 2A). A new broad resonance at ∼2.40 ppm and a singlet resonance at 2.06 ppm are indicative for in-chain ketones and ketones at the pre-terminal position, respectively. Ketone and aldehyde formation is also seen by FTIR. The two new vibrations at 1700 cm^−1^ and 1732 cm^−1^ (Fig. 2B) are in line with results for PP/CO copolymers for which ketone C=O stretching vibrations are typically seen between 1690 cm^−1^ and 1715 cm^−1^.^20^ The observed vibrations are also in line with end-capped aldehydes in PE materials as well as aldehydes installed on in-chain primary carbons in the backbone of PP.^20–25^ For PP, we obtained oxyfunctionalisation degrees of 0.13% FG_Aldehyde_ and 0.50% FG_Ketone_ and with that an overall functionalisation degree of 0.63%. Again, GPC analysis demonstrated no backbone cleavage or crosslinking, again highlighting the well-behaved reactivity of the FRP approach (Fig. 2C).

**Fig. 2:**
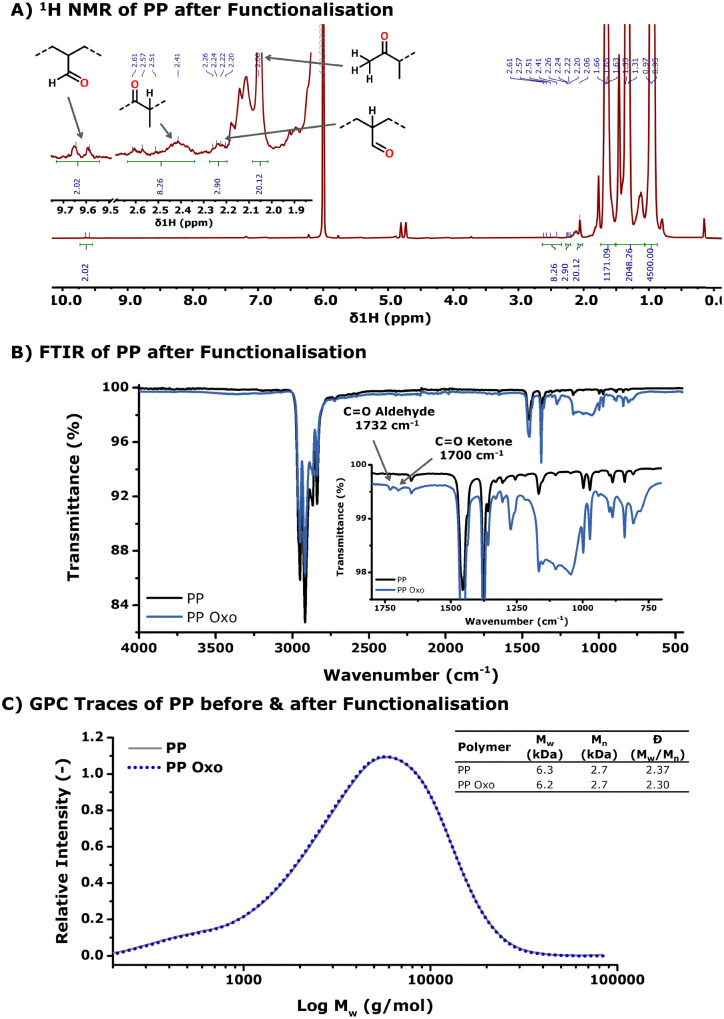
(A) ^1^H NMR spectrum of the product obtained after the oxidation reaction of PP, measured in 1,1,2,2-tetrachloroethane-d_2_ at 120 °C, (B) FTIR spectra of PP and the product obtained after the oxidation reaction of PP and (C) Gel Permeation Chromatography (GPC) traces of PP before and after oxyfunctionalisation, with the corresponding *M*_w_, *M*_n_ and *Đ*.

The thermal properties of the modified polymers were studied with TGA which showed minor changes in the decomposition temperature for both samples (Fig. 3). A 5 °C decrease in *T*_d(10 wt%)_ is in line with literature on ketone-functionalised PE.^11,12^ Oxo-PP on the other hand displayed an increase in *T*_d(10 wt%)_ of ∼12 °C; this first example of PP with a low FG_Oxo_% thus shows different behaviour to the well documented Isotactic and atactic poly(propylene-*alt*-CO) which typically exhibit a *T*_d(10 wt%)_ between 328–355 °C, 25–52 °C lower than pristine PP (∼380 °C).^37,38^ The amount of installed functional groups and the position along the backbone, *i.e.* in-chain or on a pendant position, might therefore be important parameters to tune for to control the thermal properties of PP.

**Fig. 3:**
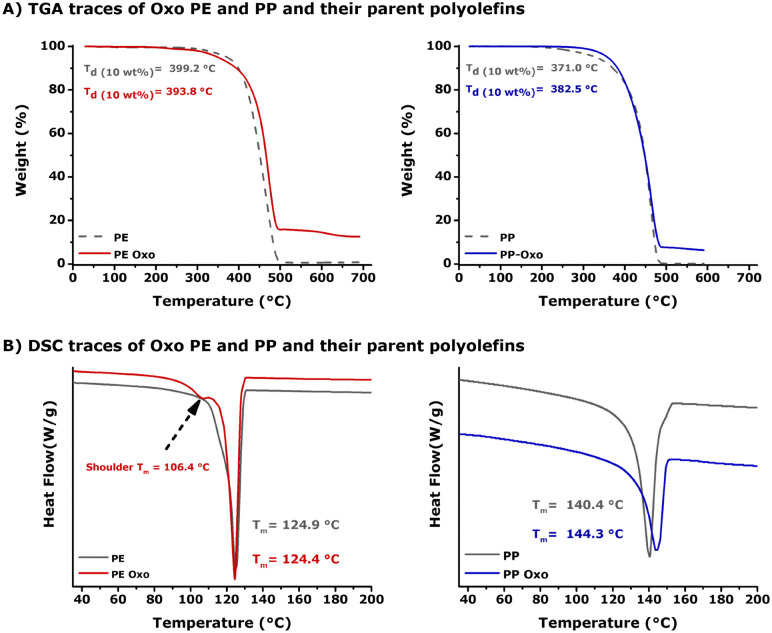
(A) TGA analysis of PE and PP and the corresponding oxyfunctionalised polyolefins after functionalisation with their respective degradation temperatures. (B) MDSC analysis of PE and PP and the corresponding oxyfunctionalised polyolefins after functionalisation with their respective melting temperatures.

Modulated differential scanning calorimetry (MDSC) measurements showed the oxo-PE melting temperature (*T*_m_ 124.4 °C) to be almost identical to PE. A shoulder melting event is also observed at 106.4 °C, which could suggest that only some of the polymer chains may be functionalised with ketones. The work of the group of Hartwig showed a linear correlation between functionalisation degree and *T*_m_ decrease and, while much smaller, the decrease seen herein is in line with this.^12^ Interestingly, we observe a small increase in *T*_m_ for the oxyfunctionalised PP from 140.4 °C to 144.3 °C, indicative of subtle changes caused by secondary effects in crystalline morphology rather than major structural modification.^39,40^ Compared to poly(propylene-*alt*-CO) copolymers, our results highlight that the instalment of a small amount of functional groups can lead to an enhancement in both the *T*_d_ and the *T*_m_, while the copolymers show a lower *T*_d_ and *T*_m_ compared to PP.^37^

This study shows that oxyfunctionalisation of PE and PP was achieved using FRP chemistry ketone or aldehyde functional groups introduction with functionalisation degrees of 0.3% for PE and 0.6% for PP. The metal-free, radical-based approach is notably well controlled, as no evidence is seen for backbone cleavage or crosslinking. Beyond oxyfunctionalisation, other chemistries can now also be explored for these polyolefins after FRP C–H bond activation.

## Conflicts of interest

There are no conflicts to declare.

## Supplementary Material

CC-062-D6CC01531J-s001

## Data Availability

The data related to the work described in this paper is available in the Yoda repository using the following link: https://doi.org/10.24416/UU01-EHMXF5. Supplementary information: experiments, oxyfunctionalisation reactions on small model compounds and polyolefin materials and NMR and FTIR spectra, ESI-MS data and thermogravimetric analysis (TGA), differential scanning calorimetry (DSC) traces and gel permeation chromatography (GPC) traces. See DOI: https://doi.org/10.1039/d6cc01531j.
