# Supplementary material for: Post-polymerisation modification of polyolefins through C–H bond activation by frustrated radical pairs
Source: Chem Commun (Camb). 2026 Jul 15. Online ahead of print. doi: 10.1039/d6cc01531j (PMC13403482; doi:10.1039/d6cc01531j)
Supplement: CC-OLF-D6CC01531J-s001 [file CC-OLF-D6CC01531J-s001.pdf]

Supplementary Information (SI) for Chemical Communications.
This journal is © The Royal Society of Chemistry 2026

Supporting Information

Post-Polymerisation Modification of Polyolefins through C-H bond Activation by Frustrated Radical Pairs

Maartje Otten[a], Jeroen Hendriks[a], Léon Witteman[a], Arnaud Thevenon[a*] and Pieter C.A. Bruijnincx[a*]

a) Organic Chemistry & Catalysis, Institute for Sustainable and Circular Chemistry, Faculty of Science, Utrecht University, The Netherlands

* Corresponding Authors: Arnaud Thevenon and Pieter C.A. Bruijnincx, Organic Chemistry & Catalysis, Institute for Sustainable and Circular Chemistry, Faculty of Science, Utrecht University, Universiteitsweg 99, 3584 CG Utrecht, The Netherlands; Email: a.a.thevenon-kozub@uu.nl; p.c.a.bruijnincx@uu.nl

Table of Contents

1. General 2
2. Synthesis 3
2.1 General procedure A: C-H oxidation of small molecule substrates 4
2.2 General procedure B: C-H oxidation of PE and PP 5
3. PE Oxyfunctionalization Results 8
4. (VT-)NMR, IR, ESI-MS Data 9
5. DSC and TGA Data 21
6. GPC Data 23
References 25

1. General

Unless stated otherwise, all functionalisation reactions were performed under N_2 atmosphere in a 8 mL glass vial with a PTFE septum cap (airtight up to 72 h). Synthesis of the polymerisation catalyst was performed in a nitrogen filled UNILAB^{plus} M. Braun glovebox according to literature.[1] Polyethylene was self-synthesised according to protocol literature with a M_w of 1900 g/mol.[1] Polypropylene (isotactic) was acquired from Sanyo (Viscol 660-P) with a M_w of 6300 g/mol. Toluene, diethyl ether and hexane were taken from an MBraun MB SPS-80 purification system dried further over 4 Å molecular sieves, degassed and tested for water content by the Karl-Fisher titration before use. Tetrahydrofuran (THF) was dried over sodium benzophenone ketyl, distilled under inert N_2(g) and tested for water content by the Karl-Fisher titration before use. Dried MeCN was collected from an M. Braun MB-SPS-800 system and was degassed by bubbling N_2 through the solvents for at least 30 min. 1,2,4-trichlorobenzene and α,α,α-trifluorotoluene were dried and stored over 4 Å molecular sieves and degassed prior to use. All reagents and starting materials were purchased from commercial sources and used without further purification, except when specified. Deuterated solvents were purchased from the Cambridge Isotope Laboratory Incorporation (Cambridge, USA) or Sigma-Aldrich and used as received. 1,2,4-trichlorobenzene, 3-chloroperoxybenzoic acid, nitrosonium tetrafluoroborate, *n*-octadecane and *n*-undecane were purchased from Acros. Cyclohexane was purchased from Boom. α,α,α-Trifluorotoluene, lithium hexamethyl-disilazane, and TEMPO were purchased from Merck. Methanol was purchased from VWR. The purity of 3-chloroperoxybenzoic acid was determined to be 60 wt% by quantitative NMR analysis.

NMR spectroscopy (^{1}H, ^{13}C{^{1}H}, ^{1}H-^{13}C and ^{19}F) was conducted using a 400 MHz Varian spectrometer equipped with AutoX probe and Agilent ProTune probe tuning accessory or a 400 MHz Jeol EZCL G spectrometer with a HFX probe. ^{1}H and ^{13}C NMR chemical shift are reported in the standard δ notation of part per million (ppm) and are referenced to a residual peak of the solvent, as determined relative to $SiMe_4$. Infrared spectroscopy was conducted using a PerkinElmer SpectrumTwo FT-IR Spectrometer equipped with an ATR-probe. Peaks are annotated by (w), (m) and (s) to indicate weak, medium and strong signals, respectively. Electron spray ionisation mass spectrometry (ESI-MS) spectra were recorded on an Advion Expression CMS mass spectrometer and are shown in positive scanning mode unless stated otherwise.

Thermogravimetric analysis (TGA) measurements were performed on a TA Instruments TGA Q50 Thermographic Analyser under nitrogen atmosphere on a platinum pan and analysed using TA Instruments Universal Analysis 2000 software. The thermal degradation was investigated with approximately 10-20 mg sample, loaded onto the pan and heated from ambient temperature to 700 °C with a rate of 20 °C/min. Modulated Differential Scanning Calorimetry (MDSC) measurements were performed on a TA Instruments Discovery DSC equipped with- a TA Instruments Refrigerated Cooling System 90 and analysed with TA Instruments Trios Software (version 5.7.1.74). Tzero low mass aluminium pans were loaded with approximately 3-5 mg of sample and closed with a Tzero aluminium lid. Samples were first kept at 25 °C and thereafter cooled down at a rate of 2.00 °C/min to -90 °C. After 10 min at -90 °C, the sample was heated to 80 °C at a rate of 2.00 °C/min. After 10 min at 80 °C, the sample was cooled down to 25 °C. The modulation was set at 1 °C/min.

Gel permeation chromatography (GPC) was performed on a Polymer Char (Valencia, Spain) GPC-IR instrument equipped with an infrared detector (IR4), three PLgel Olexis (300 × 7.5 mm, Agilent Technologies) columns in series and a PLGel Olexis (50 × 7.5 mm, Agilent Technologies) guard column. The GPC eluent used was 1,2,4-trichlorobenzene, containing 300 ppm butylated hydroxytoluene, at a flow rate of 1.0 mL/min with a column temperature of 160 °C. The PE and PP samples were prepared with a concentration of 1.0 mg/mL with heptane as an internal standard. The samples were dissolved at 160 °C under N_2 atmosphere for 90 min under continuous gentle shaking and filtered prior to injection. The molecular weight was calculated with respect to polystyrene standards (Polymer Char Laboratories, M_n = 100 g/mol up to *Mn* = 1510000 g/mol) and converted to PE and PP equivalents.

2. Synthesis

2,2,6,6-Tetramethyl-1-oxo-piperidinium tetrafluoroborate (TEMPO$^+$BF$_4^-$)

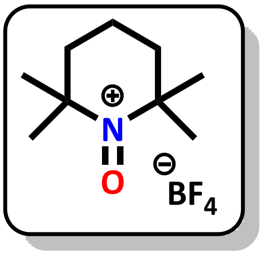

Instead of the typical protonation of TEMPO with a strong acid such as HBF_4, followed by addition of sodium hypochlorite (NaOCl) to give the oxoammonium salt, we explored a different synthesis route to obtain the TEMPO oxoammonium salt using nitrosyl tetrafluoroborate.[2–5] An oven-dried Schlenk flask loaded with a stir bar was charged with TEMPO (2.0 g, 12.11 mmol, 1.0 equiv) and dissolved in dry and degassed MeCN (20 mL), yielding a bright red solution. Quickly, $NO^+BF_4^-$ (1.46 g, 12.46 mmol, 1.02 eq.) was weighed and added to the red solution, while stirring vigorously. Instantly a lot of gas formation was observed, and the colour changed to a black/yellow colour. Over the next couple of minutes, the colour faded to a more orange-brown solution. After 15 min, the solvent was evaporated under vacuum yielding bright yellow solids. The solids were hereafter filtered and washed with ice-cold water and diethyl ether on the filter. The obtained yellow solids were collected and dried extensively under vacuum and stored at -18 °C prior to use. The title compound was obtained as a bright yellow solid in 95% yield (2.81 g).

^{1}H NMR (400 MHz, MeCN-d_3, 25 °C) δ 2.51 (br t, 4H, -CH_2-C**H_2**-C(CH_3)$_2$), 2.41 (br m, 2H, -C**H_2**-CH_2-C(CH_3)$_2$), 1.67 (br s, 12H, -CH_2-CH_2-C(C**H_3**)$_2$) ppm.

^{19}F NMR (376 MHz, MeCN-d_3, 25 °C) δ -151.41 (s), -151.53 (s) ppm.

ATR-IR 521 (w), 2969 (s, br), 1098 (m), 1382 (w), 1473 (w), 1626 (m), 2939 (w), 3001 (w)

ESI-MS (MeCN): m/z = 156.4 {[TEMPO]$^+$, calc. 156.3}

Lithium pentamethylcyclopentadienyl (LiCp)*[1,6]

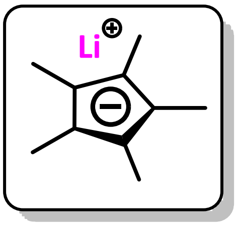

Pentamethylcyclopentadiene (2 g; 14.7 mmol) was dissolved in dry and degassed hexane (70 mL) under a N_2 atmosphere in a Schlenk flask. The reaction mixture was cooled down to -78 °C using a dry ice/acetone bath whereafter *n*-butyllithium (1.6 M in hexane) (9.6 mL; 15.4 mmol) was added dropwise. After stirring the reaction mixture for 30 min at -78 °C, the reaction mixture was allowed to warm up to ambient temperature and the colourless solution was left to stir for 16 h. The solids were washed twice with 10 mL dry and degassed hexane. The obtained white solids after filtration were dried in *vacuo* and stored under N_2 atmosphere. The title compound was obtained as a white solid (1.76 g; 84%).

Lithium bis(pentamethylcyclopentadienyl)dichlorobis(diethyl ether)neodymate(III)[1,7]

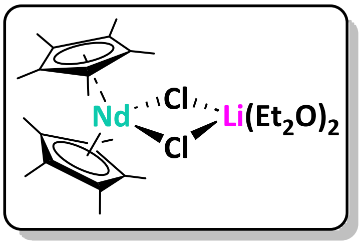

Neodymium trichloride (176 mg; 0.7 mmol) and lithium pentamethylcyclopentadienyl (201 mg; 1.4 mmol) were dissolved in dry and degassed THF (7.5 mL) under a N_2 atmosphere in a Schlenk. The brown suspension was refluxed for 16 h, whereafter the mint green suspension was allowed to cool down to ambient temperature. The THF was removed in *vacuo* and the remaining mint green solids were washed twice with dry and degassed diethyl ether (5 mL). The white solids were filtered off, whereafter a green/blue ether extract was obtained and cooled to -40 °C to yield large dark blue crystals. The title compound was obtained as dark blue solids (269 mg; 60%).

Polyethylene

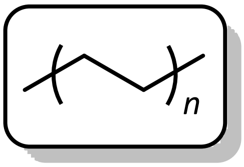

Lithium bis(pentamethylcyclopentadienyl)dichlorobis(diethyl ether)neodymate(III) (2.6 mg; 4.1 μmol) was mixed with dry and degassed toluene (10 mL). di-*n*-Butyl magnesium (0.5 M in heptane) (0.4 mL; 0.2 mmol) was added to the mixture and stirred for 30 min. To a 500 mL Schlenk flask, dry and degassed toluene (20 mL) was added whereafter the toluene was freeze-pump-thawed before applying 1 bar of ethylene. The Schlenk flask with 1 bar of ethylene was heated to 80 °C, whereafter the 10 mL neodymium catalyst solution with di-*n*-butyl magnesium was added to the Schlenk. With a manometer the ethylene flow was monitored and after 30 min the reaction was cooled down to ambient temperature. The reaction mixture was thereafter quenched with methanol (150 mL) while vigorously stirring. The white solid flakes formed were thereafter filtered and washed extensively with methanol. The obtained white solids were dried in a vacuum oven at 75 °C for 24 h, whereafter the solids were grinded with a mortar and pestle. The title compound was obtained as a white powder (1.6 g).

^{1}H NMR (400 MHz, Toluene-*d8*, 100 °C) δ = 1.34 (s, 357H, -C**H_2**-), 0.89 (t, 6H, -C**H_3**) ppm.

^{13}C NMR (101 MHz, Toluene-*d8*, 100 °C) δ = 14.3, 30.0, 30.5, 32.6 ppm.

2.1 General procedure A: C-H oxidation of small molecule substrates

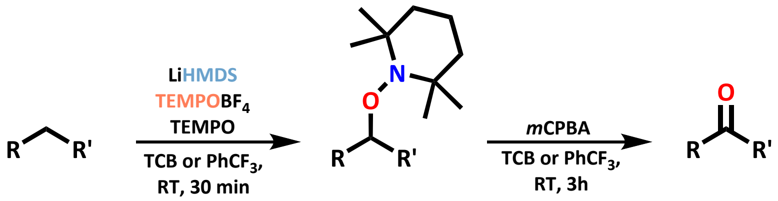

An oven-dried 8 mL vial containing a stir bar was loaded with the substrate (in case of solid) (2.0 mmol), $TEMPO^+BF_4^-$ (48.7 mg, 0.2 mmol, 1.0 equiv) and TEMPO (6.3 mg, 0.04 mmol, 0.2 equiv). The vial was capped with a septum and to it was added dried and degassed solvent (1.0 mL) and substrate (in case of liquid) (2.0 mmol). The headspace of the vial was purged with N_2 for 5 min. In a glovebox, LiHMDS (41.3 mg, 0.25 mmol, 1.25 equiv) was weighed in an 8 mL vial and capped with a septum. Outside of the glovebox dried and degassed solvent (1.0 mL) was added to dissolve the LiHMDS. The resulting crème coloured solution was added dropwise to the yellow suspension containing the substrate while stirring vigorously. The reaction mixture was stirred at ambient temperature for 30 min. During this time, the mixture slowly changed to an orange/brown colour, indicating consumption of the $TEMPO^+BF_4^-$. After this, 20 μL of H_2O was added to quench any remaining LiHMDS. A solution of *m*-CPBA (63.3 mg, 0.22 mmol, 1.1 equiv) in DCM (2.0 mL) was made and added dropwise to the reaction mixture, turning it into an orange suspension. This was left to stir for 3 h at ambient temperature. Afterwards, the reaction mixture was added to a saturated solution of Na_2SO_3 in H_2O (10 mL). Work-up was done by extracting the obtained two-layer system of solvent and H_2O with DCM (3 x 10 mL), after which the combined organic layers were washed with brine (20 mL). The obtained slightly orange-coloured organic layer was dried using Na_2SO_4 and filtered before solvent evaporation on a Rotavap.

2.2 General procedure B: C-H oxidation of PE and PP

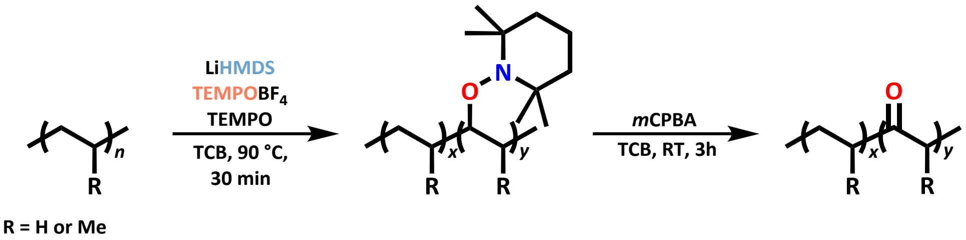

An oven-dried 8 mL vial was loaded with polymer before capping it with a septum and purging with N_2 for 3 min. Dried and degassed solvent (1.0 mL) was added and heated to 90 °C until all polymer was dissolved. After the solution was cooled to ambient temperature, the septum was removed and under an outflow of N_2 both $TEMPO^+BF_4^-$ (48.7 mg, 0.2 mmol, 1.0 equiv) and TEMPO (6.3 mg, 0.04 mmol, 0.2 equiv) were added. Again, the vial was capped with a septum and purged with N_2 for 5 min. In a glovebox, LiHMDS (41.3 mg, 0.25 mmol, 1.25 equiv) was weighed in an 8 mL vial and capped with a septum. Outside of the glovebox dried and degassed solvent (1.0 mL) was added to dissolve the LiHMDS. The resulting crème coloured solution was added dropwise to the yellow suspension containing the polymer. This mixture was heated to 90 °C in an aluminium block for 30 min, during which it turned to a clear brown colour. After letting the mixture cool down, 20 µL of H_2O was added to quench any remaining LiHMDS. A solution of *m*-CPBA (63.3 mg, 0.22 mmol, 1.1 equiv) was prepared in the same solvent (2.0 mL) and added dropwise to the reaction mixture. This was left to stir for 3 h at ambient temperature. Afterwards, the reaction mixture was added to a saturated solution of Na_2SO_3 in H_2O (10 mL). The top layer was decanted and the remaining orange/brown coloured suspension was added slowly to 30 mL of vigorously stirred methanol. The mixture was centrifuged before filtration over a Büchner funnel with filter paper. The residue was washed with plenty of H_2O and MeOH before being collected and dried extensively in a vacuum oven overnight.

Oxidation of Cyclohexane

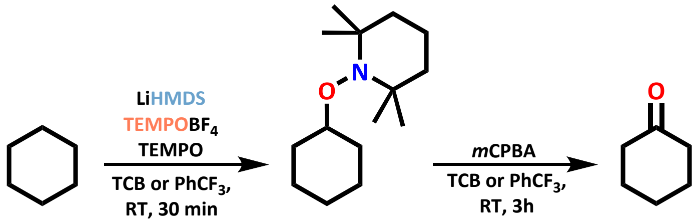

Following general **procedure A**, cyclohexane (0.22 mL, 2.04 mmol, 10 equiv) was oxidised to cyclohexanone using either 1,2,4-trichlorobenzene or α,α,α-trifluorotoluene as solvents. A crude orange-coloured mixture was obtained after extraction, which was analysed using ^{1}H NMR and FTIR spectroscopy.

^{1}H NMR (400 MHz, $CDCl_3$, 25 °C) δ 2.36 (t, $^3J_{H,H}$ = 6.6 Hz, 4H, -C**H$_2$**-C=O-C**H$_2$**-), 1.89 (p, $^3J_{H,H}$ = 6.1 Hz, 4H, -C=O-CH$_2$-C**H$_2$**), 1.78 – 1.71 (m, 2H, C=O-CH$_2$-CH$_2$-C**H$_2$**) ppm.

ATR-IR 656 (w), 693 (m), 769 (m), 1026 (m), 1067 (s), 1120 (s), 1156 (m), 1173 (m), 1321 (s), 1457 (w), 1611 (w), 1715 (w), 2942 (w) cm^{-1}.

Oxidation of n-undecane

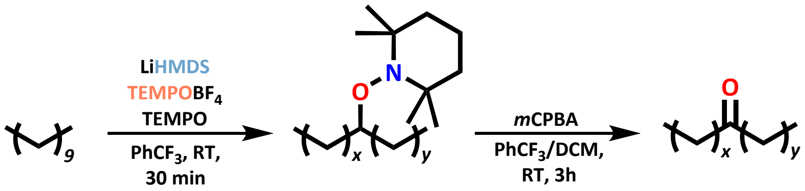

Following general **procedure A**, *n*-undecane (0.42 mL, 1.99 mmol, 10 equiv) was oxidised to *n*-undecanone using α,α,α-trifluorotoluene as solvent. A crude orange-coloured mixture was obtained after extraction, which was analysed using ^{1}H NMR and IR spectroscopy.

^{1}H NMR (400 MHz, $CDCl_3$, 25 °C) δ 2.60 – 2.31 (m, 4H, -C**H$_2$**-C=O-C**H$_2$**-), 2.15 (s, 3H, C**H$_3$**-C=O-), 1.67 – 1.56 (m, 4H), 1.29 (s, 8H, -C**H$_2$**-), 0.90 (t, $^3J_{H,H}$, 6H, C**H$_3$**-CH_2-) ppm.

ATR-IR 657 (w), 694 (m), 769 (m), 1026 (m), 1069 (s), 1126 (s), 1157 (m), 1174 (m), 1322 (s), 1457 (w), 1611 (w), 1718 (w), 2855 (m), 2925 (s), 2958 (m) cm^{-1}.

Oxidation of n-octadecane

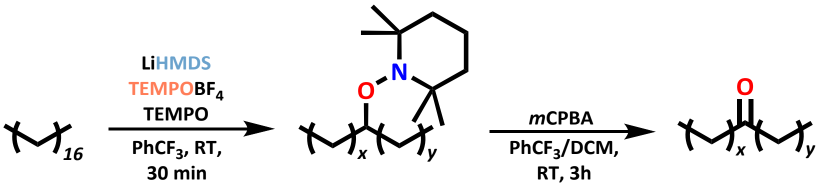

Following general **procedure A**, *n*-octadecane (499.6 mg, 1.96 mmol, 10 equiv) was oxidised to *n*-octadecanone using α,α,α-trifluorotoluene as solvent. A crude orange-coloured mixture was obtained after extraction, which was analysed using ^{1}H NMR and IR spectroscopy.

^{1}H NMR (400 MHz, $CDCl_3$, 25 °C) δ 2.44 (t, $^3J_{H,H}$ = 6.1 Hz, 4H, -C**H$_2$**-C=O-C**H$_2$**-), 2.19 (s, 3H, C**H$_3$**-C=O-), 1.86 – 1.59 (m, 4H, -C**H$_2$**-), 1.33 (s, 24H, -C**H$_2$**-), 0.95 (t, $^3J_{H,H}$ = 6.7 Hz, 6H, C**H$_3$**-C**H$_2$**-) ppm.

ATR-IR 657 (w), 693 (m), 768 (m), 1026 (m), 1069 (s), 1126 (s), 1157 (m), 1173 (m), 1322 (s), 1457 (w), 1611 (w), 1720 (s), 2854 (m), 2924 (s), 2958 (m) cm^{-1}.

Oxidation of polyethylene (PE)

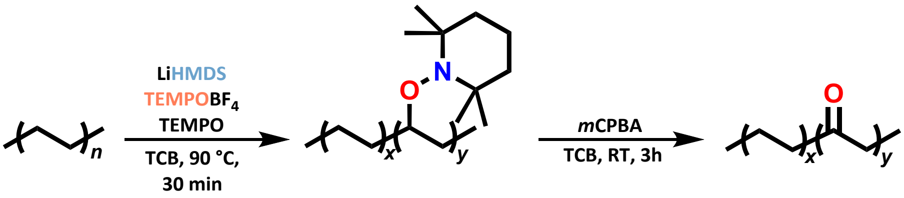

Following general **procedure B** on twice the scale, polyethylene (11.2 mg, 0.40 mmol, 1.0 equiv) was oxidised to polyethylene-ketone using 1,2,4-trichlorobenzene as solvent. After work-up a white coloured solid was obtained in 89% yield wt%.

^{1}H NMR (400 MHz, $C_2D_2Cl_4$, 120 °C) δ 5.82 (ddt, 1H, CH_2=C**H**-), 5.02 (dd, 2H, C**H$_2$**=CH-), 3.72 (s, 3H, C**H$_3$**-O-C=O-), 2.41 (t, 4H, -C**H$_2$**-C=O-C**H$_2$**-), 2.18 (s, 3H, C**H$_3$**-C=O-), 2.12 (q, 2H, -C**H$_2$**-C=O-O-), 1.60-1.20 (br s, 2H, -C**H$_2$**-) 0.96 (t, 3H, C**H$_3$**-CH_2-) ppm.

ATR-IR 719 (s), 731 (s), 910 (w), 1108 (br), 1462 (s), 1470 (s), 1640 (w), 1720 (m), 2647 (m) 2849 (s), 2916 (s) cm^{-1}.

Oxidation of polypropylene (PP)

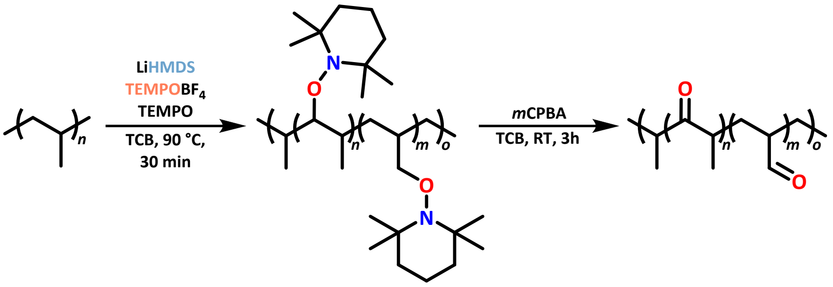

Following general **procedure B** on twice the scale, polypropylene (16.6 mg, 0.394 mmol, 1.0 equiv) was oxidised to polypropylene-ketone using 1,2,4-trichlorobenzene as solvent. After work-up a white coloured solid was reobtained in 88% yield wt%.

^{1}H NMR (400 MHz, $C_2D_2Cl_4$, 120 °C) δ 9.65-9.58 (d, 1H, **H**C=O-CH(CH_2)$_2$-), 4.81 and 4.73 (s, 2x 1H, C**H₂**-C(CH_3)-CH_2), 2.61-2.31 (m, 1H, -C=O-C**H**(CH_3)-CH_2-), 2.24 (m, 1H, HC=O-C**H**(CH_2)-), 2.06 (s, 3H, C**H₃**-C=O-CH(CH_3)-), 1.65 (br m, 1H, -CH_2-C**H**(CH_3)-), 1.40 – 1.28 (br m, 2H, -C**H₂**-CH(CH_3)-), 0.96 (br d, 3H, -CH_2-CH(C**H₃**)-) ppm.

ATR-IR 808 (m), 841 (m), 886 (m), 899 (m), 974 (m), 997 (m), 1044 (br, s), 1102 (br, s), 1168 (br, s), 1273 (m), 1303 (w), 1329 (w), 1358 (m), 1376 (s), 1436 (m), 1454 (s, br), 1652 (w), 1700 (w), 1732 (w), 2723 (w), 2839 (s), 2867 (s), 2877 (s), 2917 (s), 2950 (s), 2960 (s) cm^{-1}.

3. PE Oxyfunctionalization Results

Table S1: *Investigation of the Reaction Conditions for the C-H Bond Activation and Subsequent Oxidation of PE.*

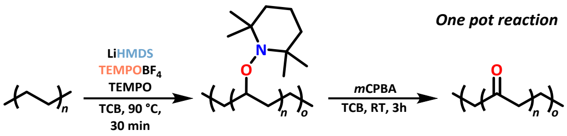

Entry	Monomer loading	Temperature C-H activation (°C)	Reaction time C-H activation (h)	Temperature oxidation (°C)	Reaction time oxidation (h)	FG_{Total} (%)	Yield recovered material (%)
1	1	90	0.5	RT	3	0.33	89
2	10	90	0.5	RT	3	0.10	93
3	5	90	0.5	RT	3	0.09	91
4	0.5	90	0.5	RT	3	0.19	90
5	1	90	18	RT	3	0.0	94
6	1	90	0.5	RT	18	0.0	88
7	1	90	0.5	90	3	0.0	93
8[a]	1	90	0.5	RT	3	0.0	92
9[b]	1	n.a.	n.a.	RT	3	0.0	89
10[c]	1	90	0.5	n.a.	n.a.	0.0	91
11[d]	1	90	0.5	RT	3	0.0	93

[a]Reaction was performed in absence of LiHMDS. [b]Reaction was performed without C-H bond activation step and only the oxidation reaction between PE and *m*CPBA. [c]Reaction was performed without the addition of *m*CPBA after the C-H bond activation. [d]Reaction was performed in the presence of 12-crown-4 (1:1 with respect to LiHMDS); n.a.: not applicable.

4. (VT-)NMR, IR, ESI-MS Data

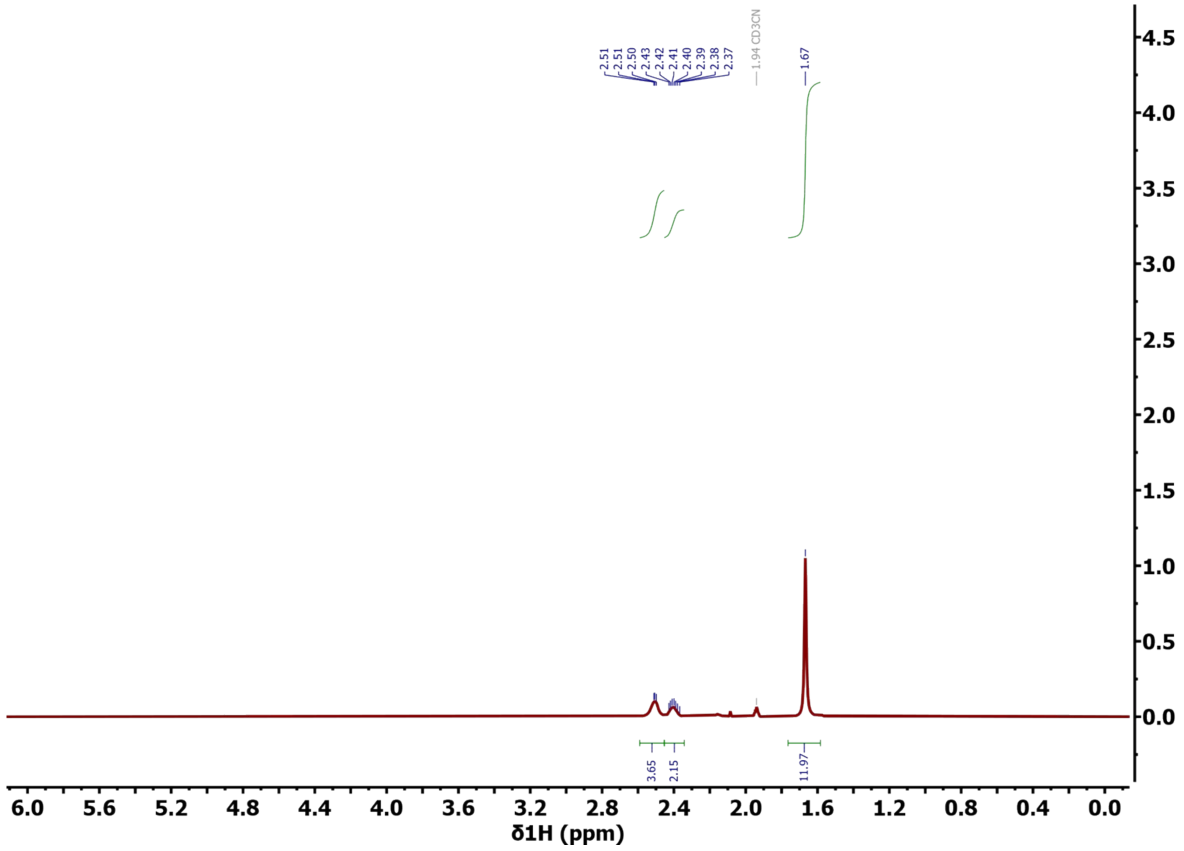

Figure S1: *^{1}H NMR spectrum of TEMPOBF$_4$, measured in CD$_3$CN at 25 °C.*

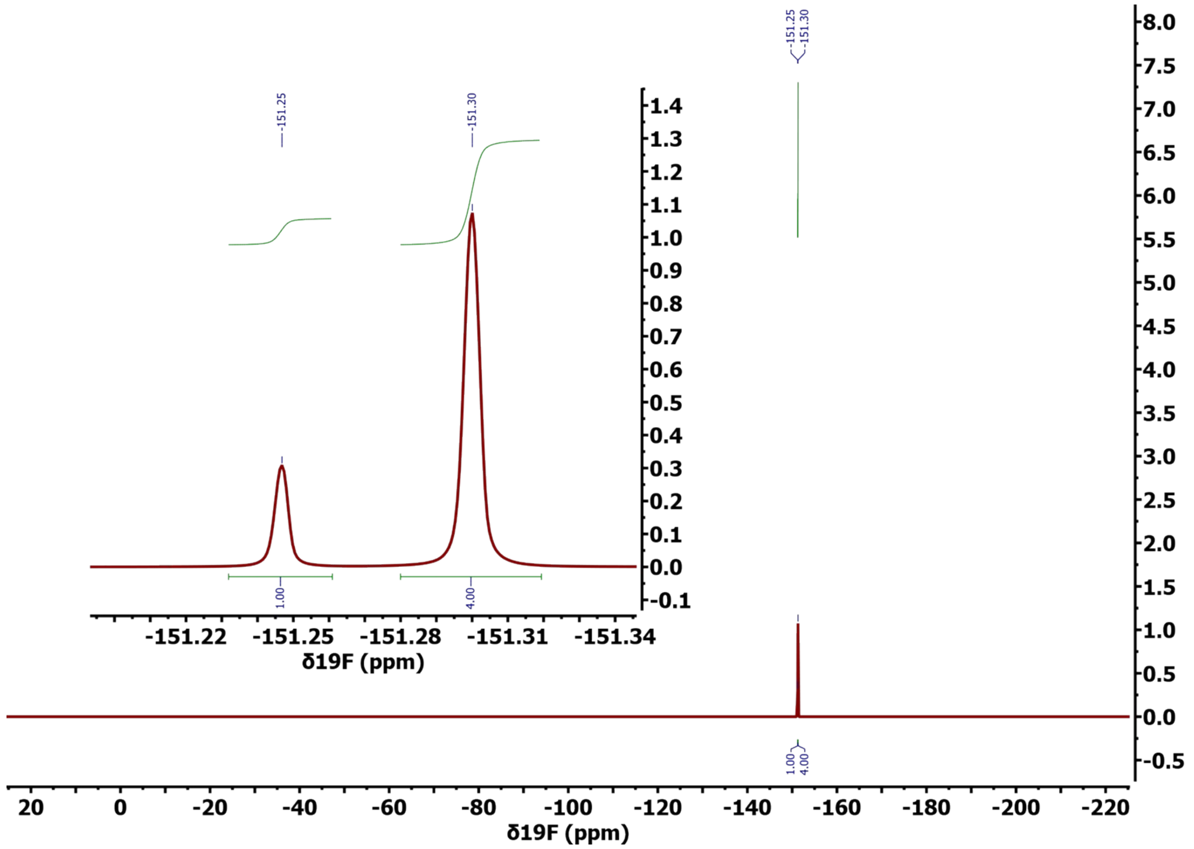

Figure S2: *^{19}F NMR spectrum of $TEMPOBF_4$, measured in CD_3CN at 25 °C.*

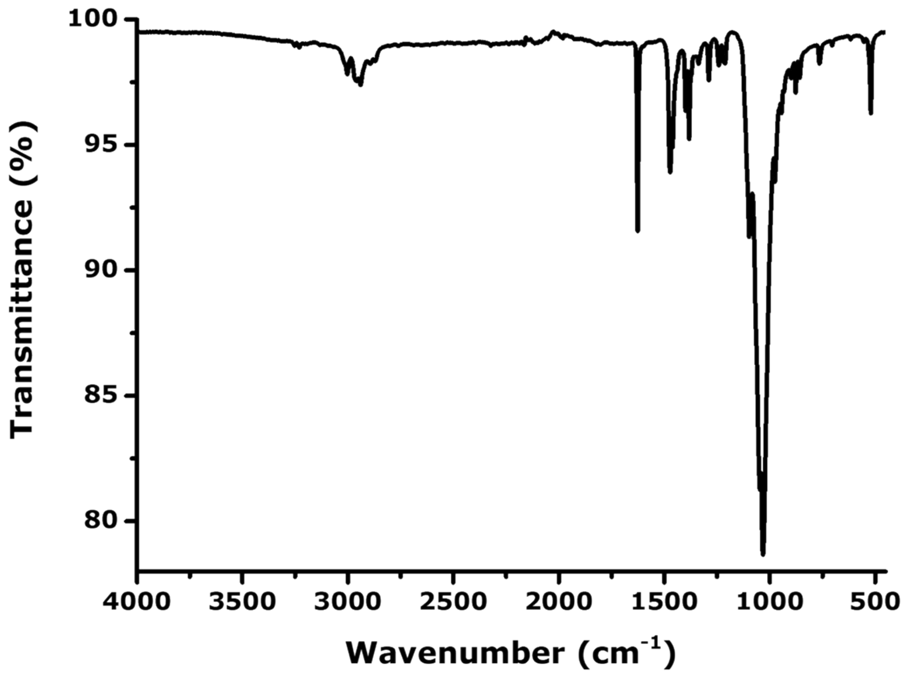

Figure S3: *FTIR (ATR) spectrum of $TEMPOBF_4$.*

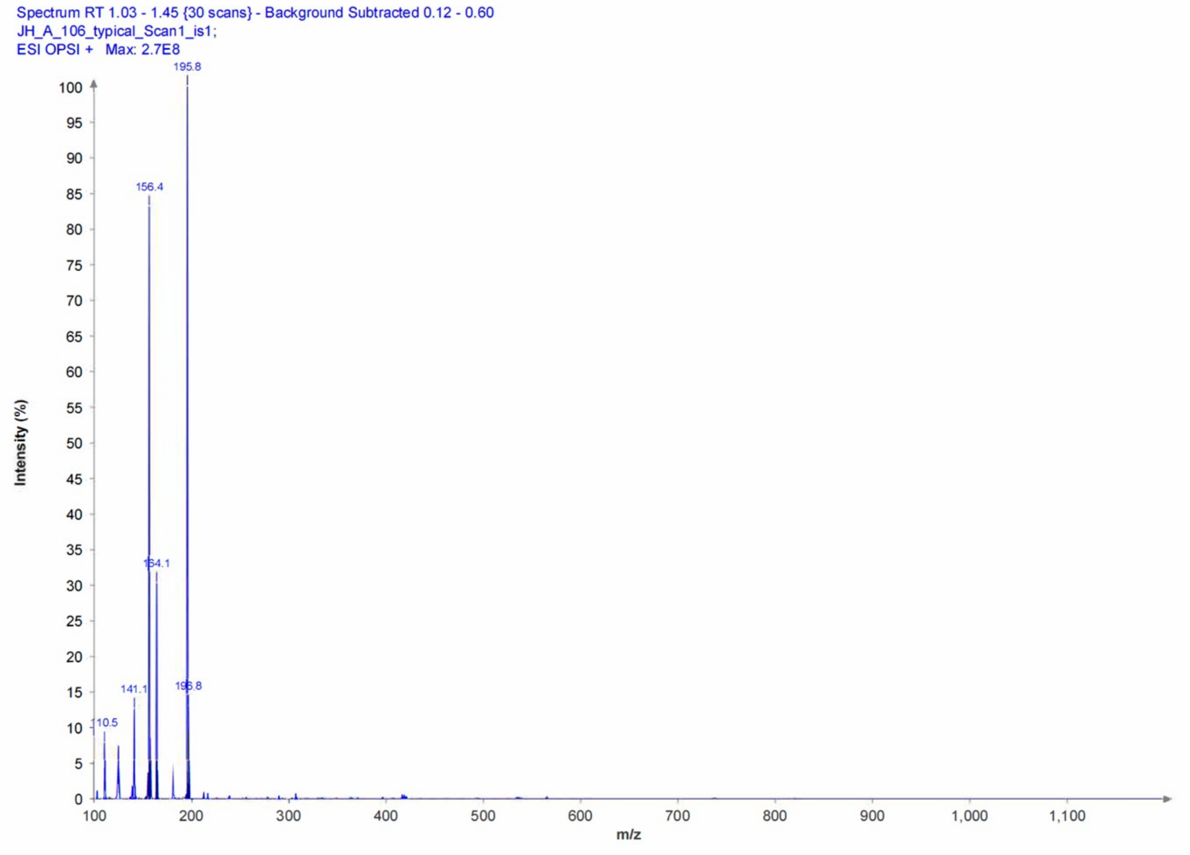

Figure S4: *ESI-MS spectrum of TEMPOBF$_4$ measured in MeCN.*

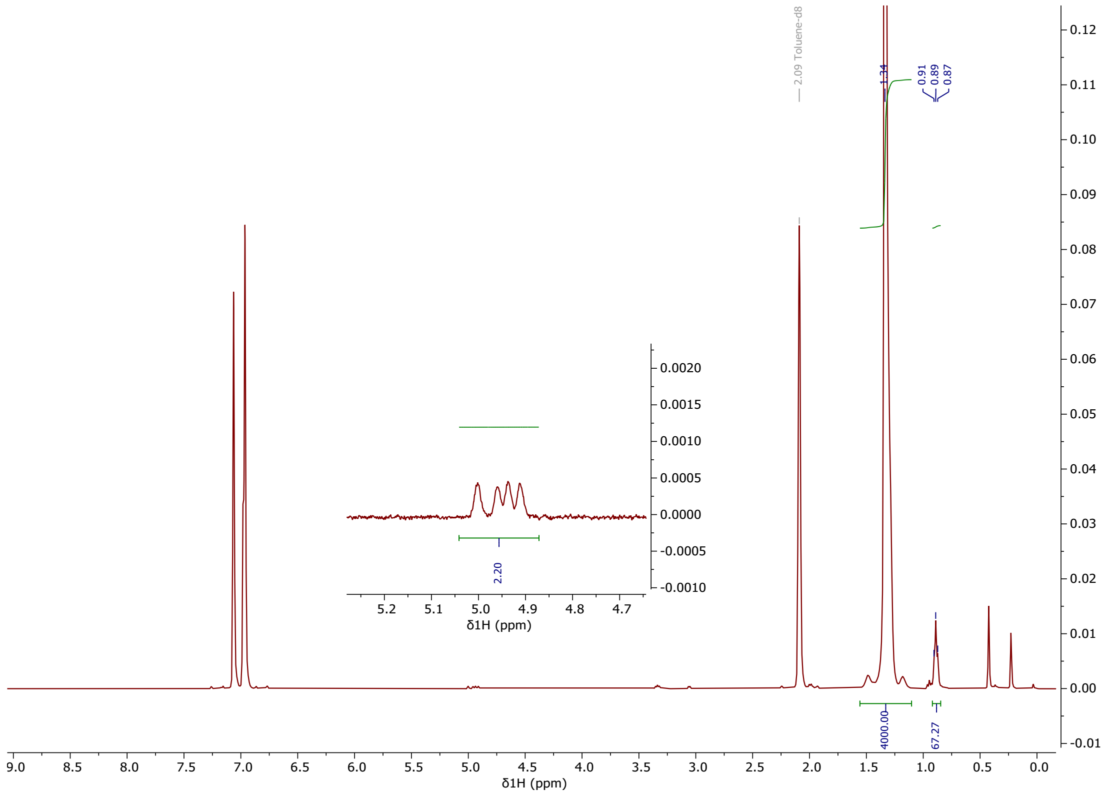

Figure S5: *^{1}H NMR spectrum of polyethylene in Toluene-d8 at 100 °C, resonances at 3.1 ppm and 3.3 ppm are NMR solvent impurities.*

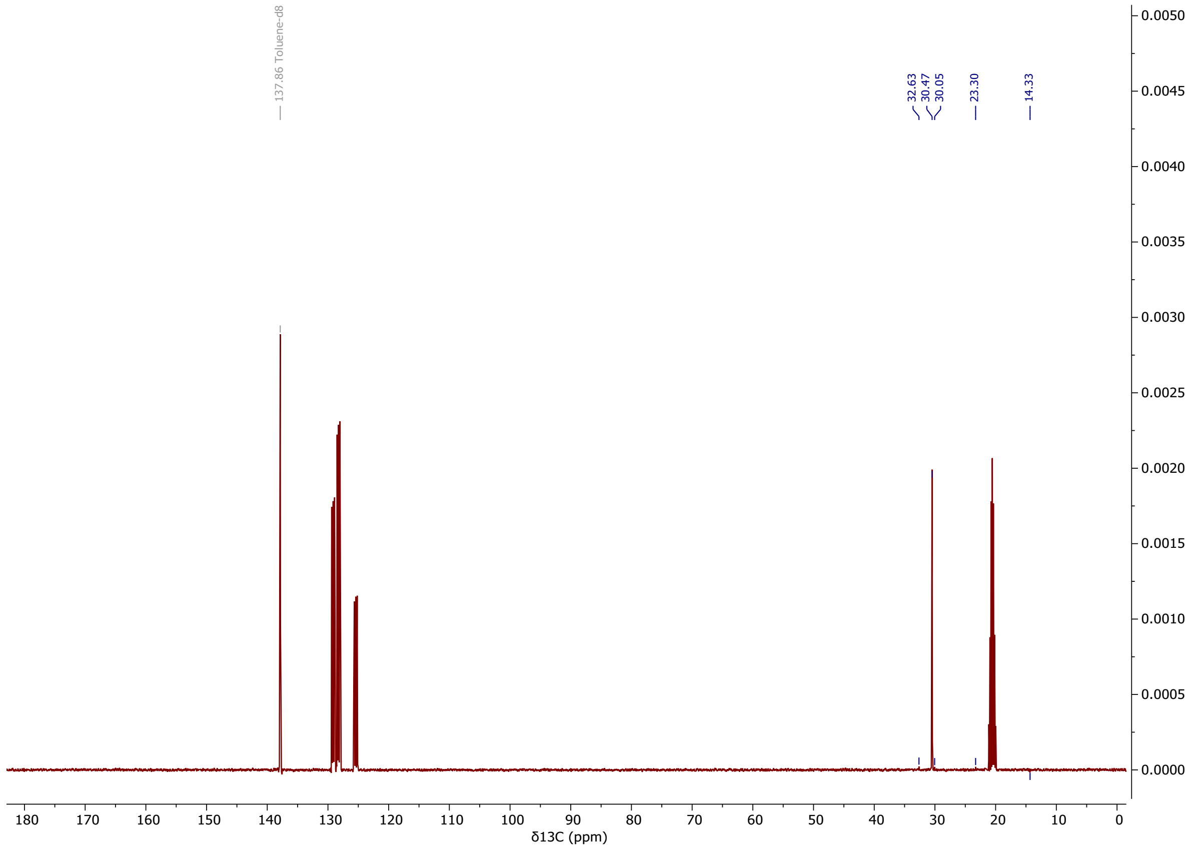

Figure S6: *^{13}C NMR (APT) spectrum of polyethylene in Toluene-d8 at 100 °C.*

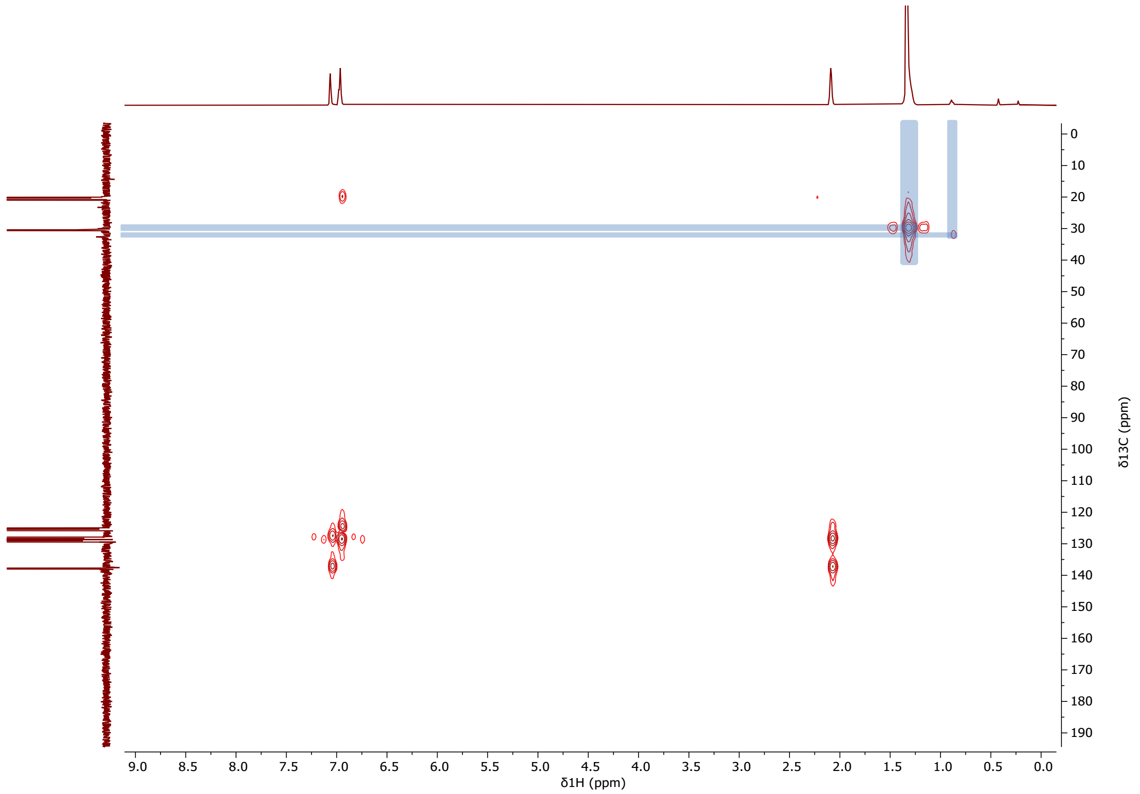

Figure S7: *^{1}H-^{13}C Heteronuclear Multiple Bond Correlation (HMBC) spectrum of polyethylene in Toluene-d8 at 100 °C.*

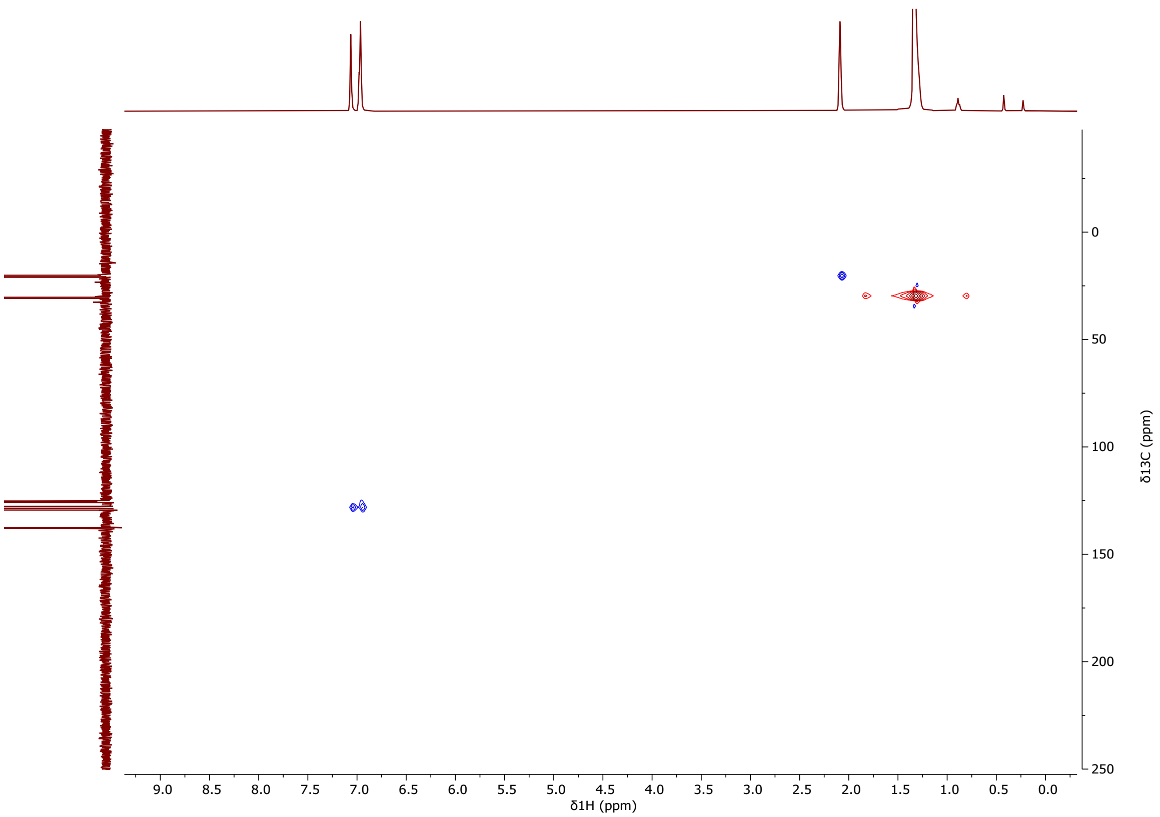

Figure S8: *^{1}H-^{13}C Heteronuclear Single Quantum Coherence (HSQC) spectrum of polyethylene in Toluene-d8 at 100 °C.*

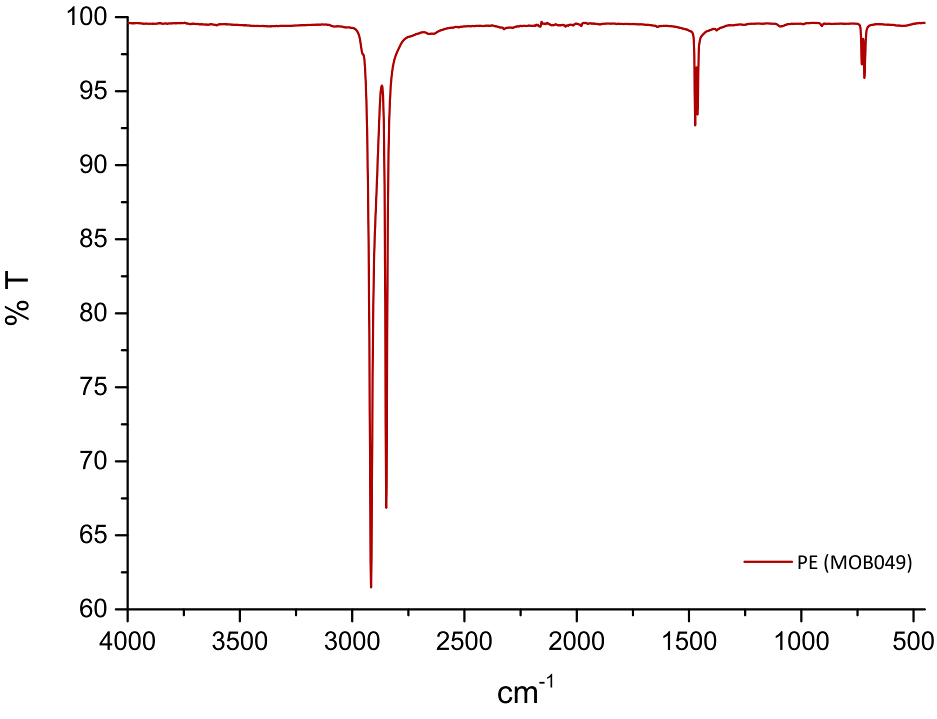

Figure S9: *FTIR (ATR) Spectrum of polyethylene.*

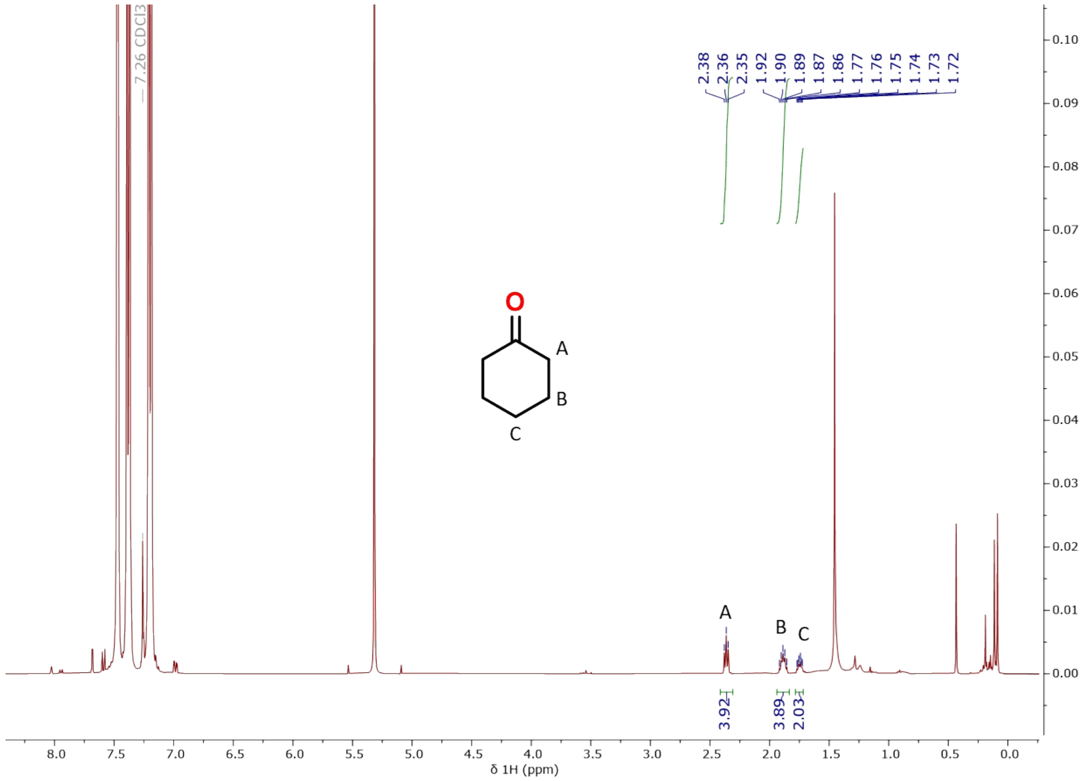

Figure S10: *^{1}H NMR spectrum after the C-H bond oxidation of cyclohexane in 1,2,4-trichlorobenzene and measured in $CDCl_3$ at 25 °C. Residual solvent peak of DCM at 5.3 ppm.*

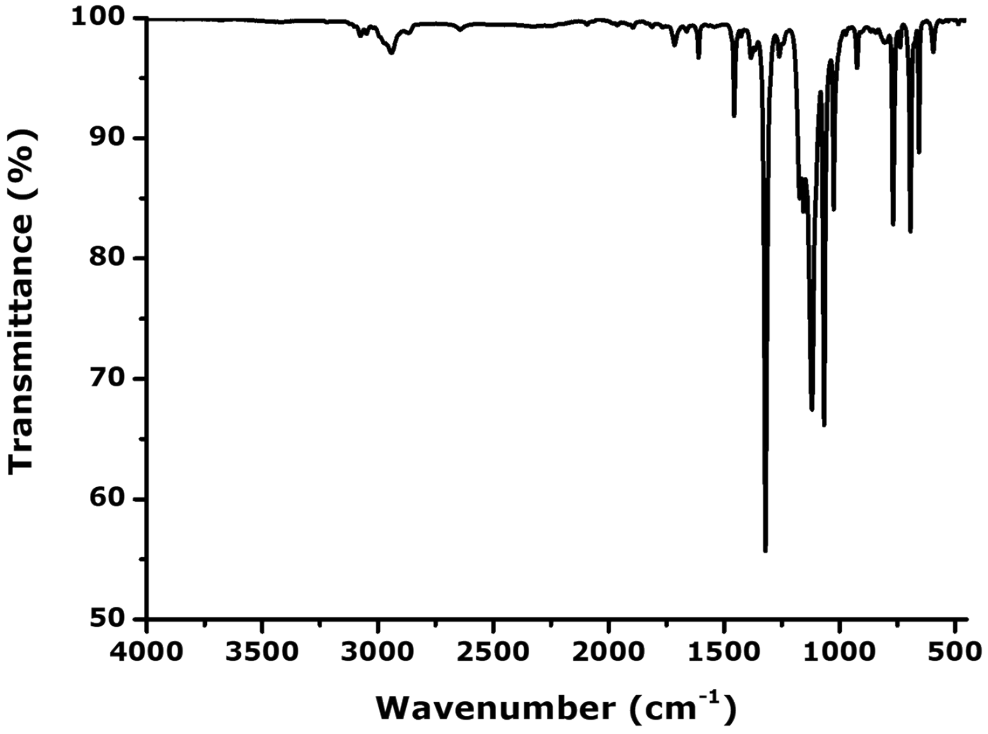

Figure S11: *FTIR (ATR) Spectrum after the C-H bond oxidation of cyclohexane in 1,2,4-trichlorobenzene.*

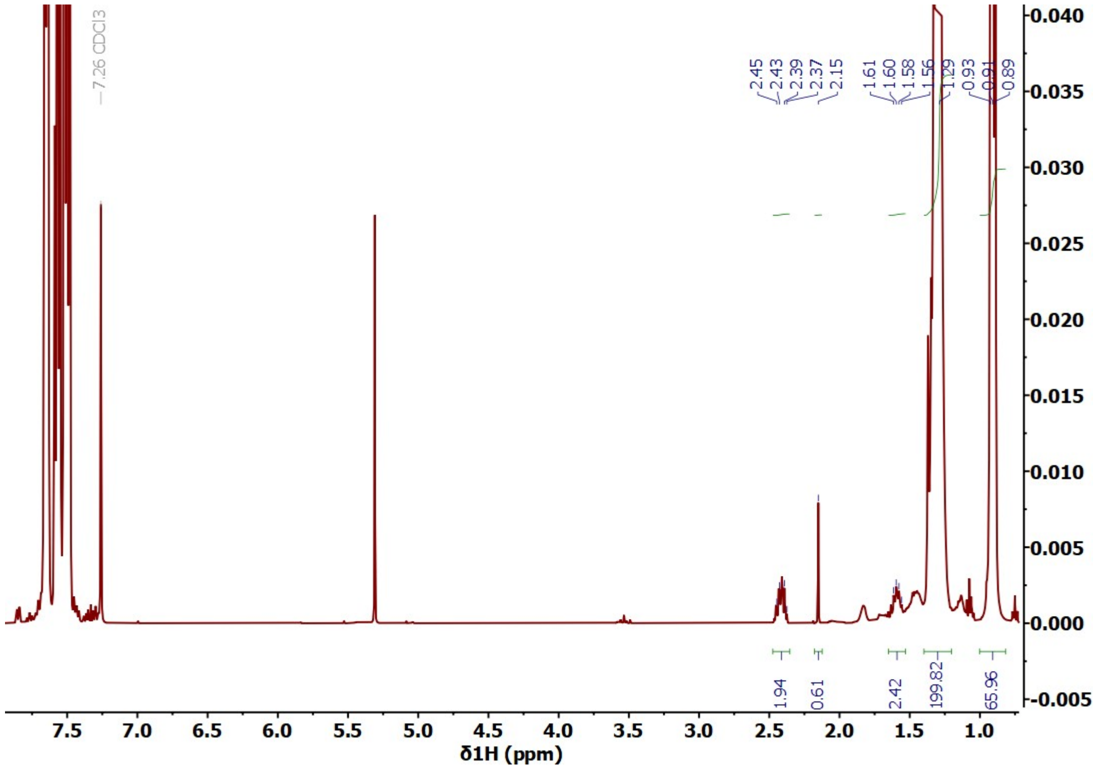

Figure S12: *^1H NMR spectrum after the C-H bond oxidation of n-undecane in trifluorotoluene and measured in $CDCl_3$ at 25 °C, with a zoom in. Peak at 5.3 ppm is residual DCM and peak around 3.5 ppm is thermal decomposition of the TEMPO BF_4 during the reaction as demonstrated by VT NMR.*

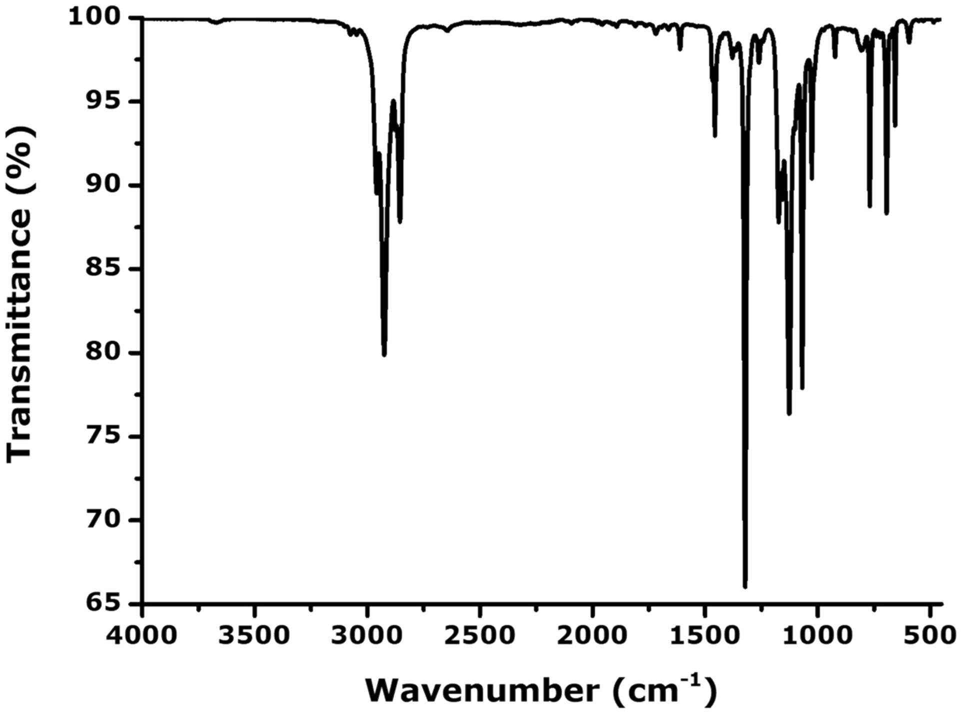

Figure S13: *FTIR (ATR) Spectrum after the C-H bond oxidation of n-undecane in trifluorotoluene.*

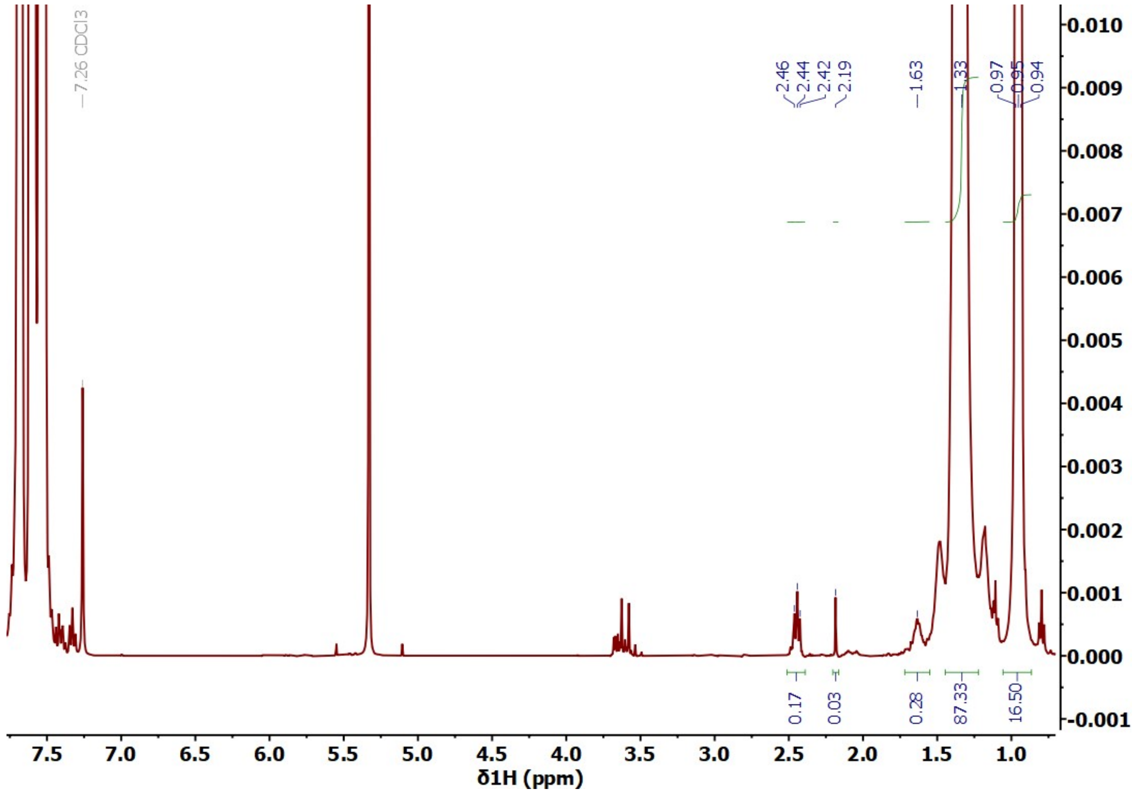

Figure S14: *^{1}H NMR spectrum after the C-H bond oxidation of n-octadecane in trifluorotoluene and measured in $CDCl_3$ at 25 °C, with a zoom in. Peak at 5.3 ppm is residual DCM and peak around 3.5 ppm is thermal decomposition of the TEMPO BF_4 during the reaction as demonstrated by VT NMR.*

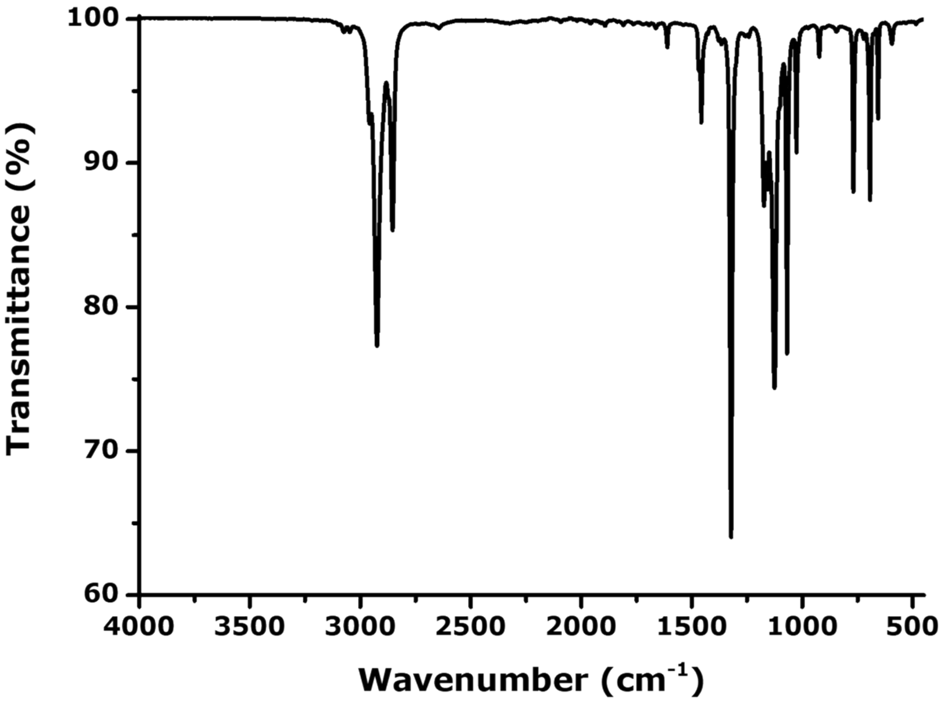

Figure S15: *FTIR (ATR) Spectrum after the C-H bond oxidation of n-octadecane in trifluorotoluene.*

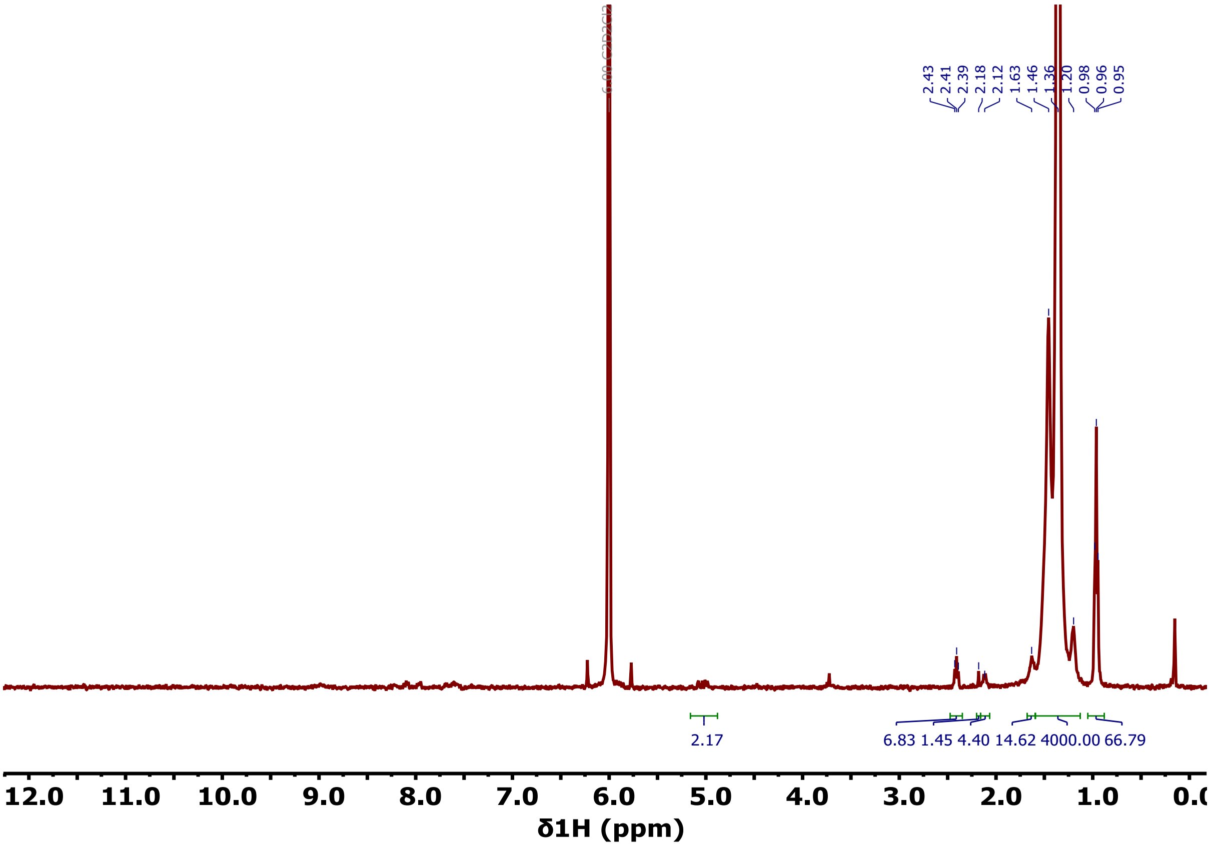

Figure S16: *^{1}H NMR spectrum of oxyfunctionalised polyethylene in $C_2D_2Cl_4$ at 120 °C, resonances at 7.5 to 8.2 ppm are of residual 1,2,4-trichlorobenzene.*

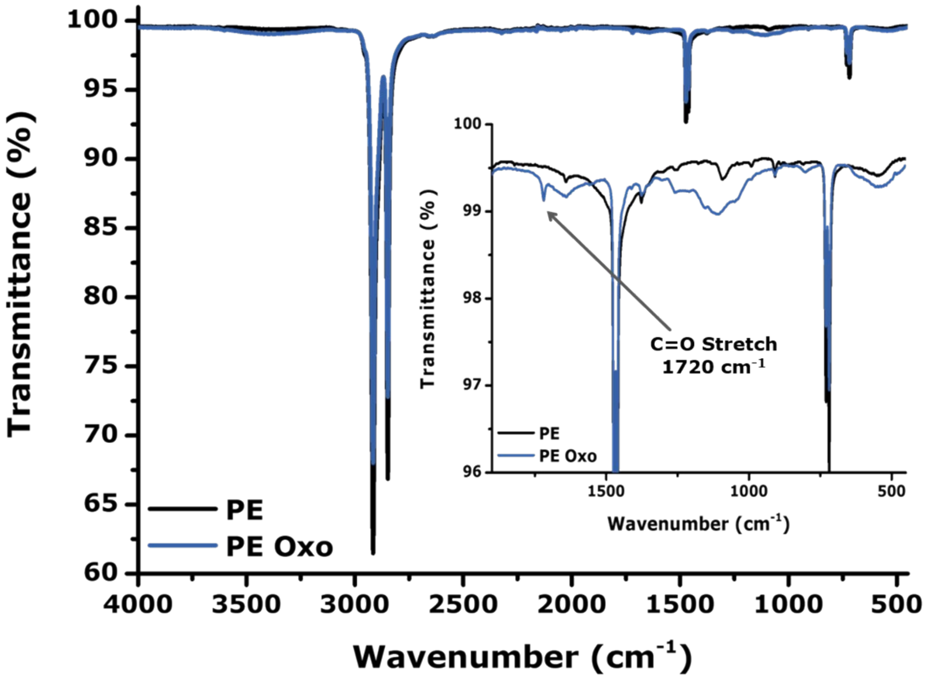

Figure S17: *FTIR (ATR) Spectrum of oxyfunctionalised polyethylene (blue) and its parent PE material (black).*

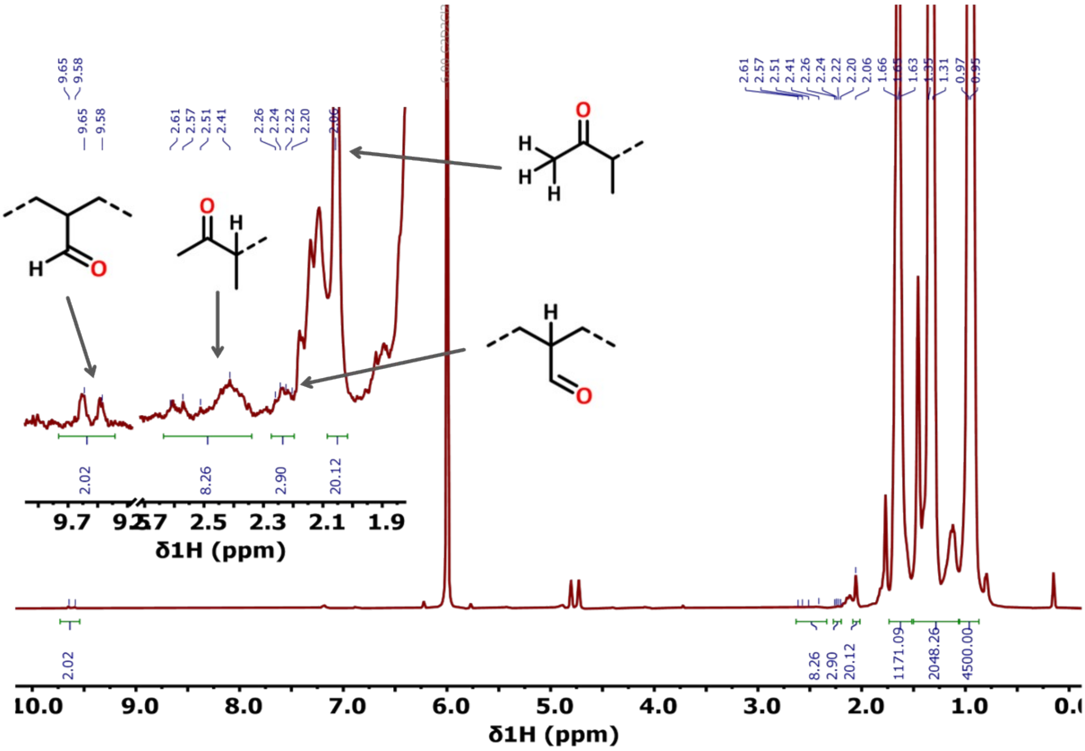

Figure S18: *^{1}H NMR spectrum of oxyfunctionalised polypropylene in $C_2D_2Cl_4$ at 120 °C.*

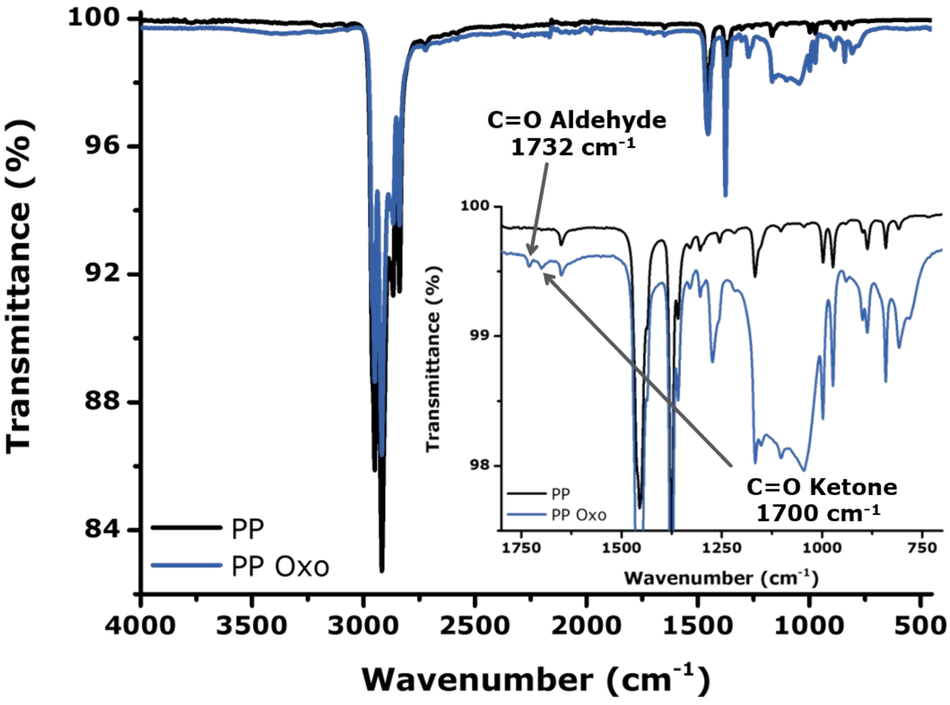

Figure S19: *FTIR (ATR) Spectrum of oxyfunctionalised polypropylene (blue) and its parent PP material (black).*

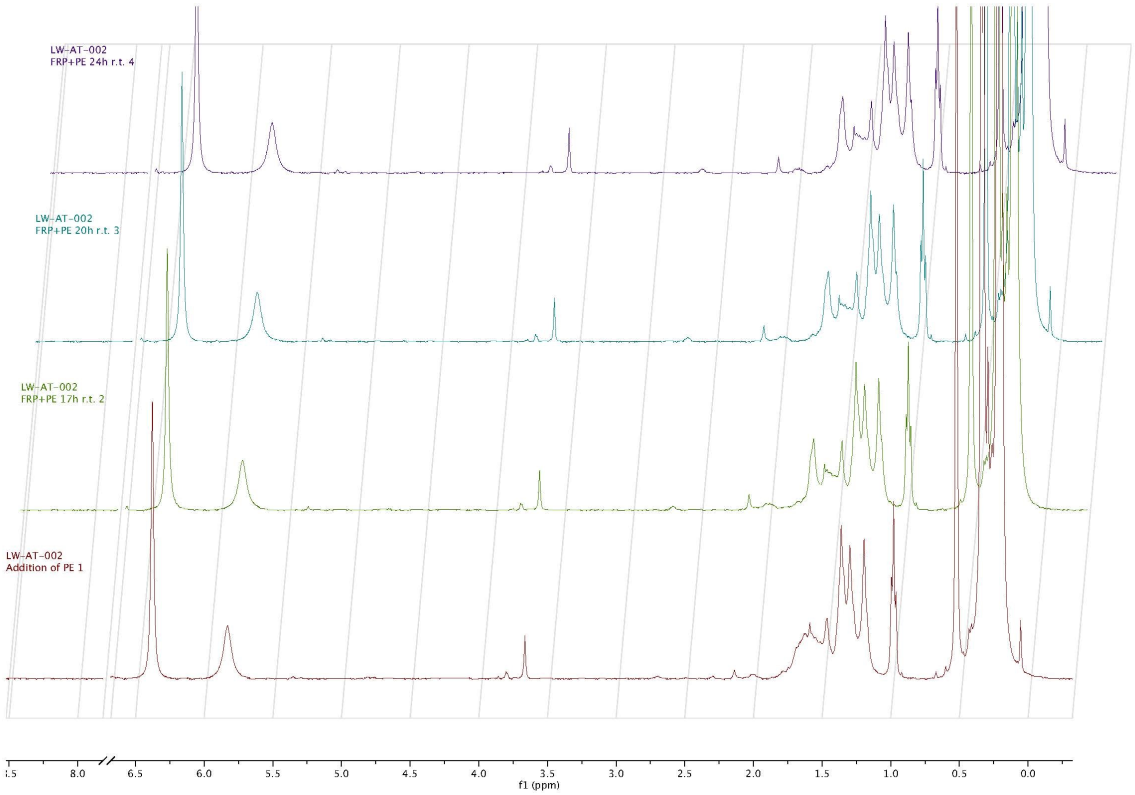

Figure S20: *VT NMR study on the stability of the FRP in the presence of PE, conducted from 30 °C up to 90 °C with 10 °C temperature differences, with TCE as internal standard in 1,1,2,2-tetrachloroethane-d$_2$.*

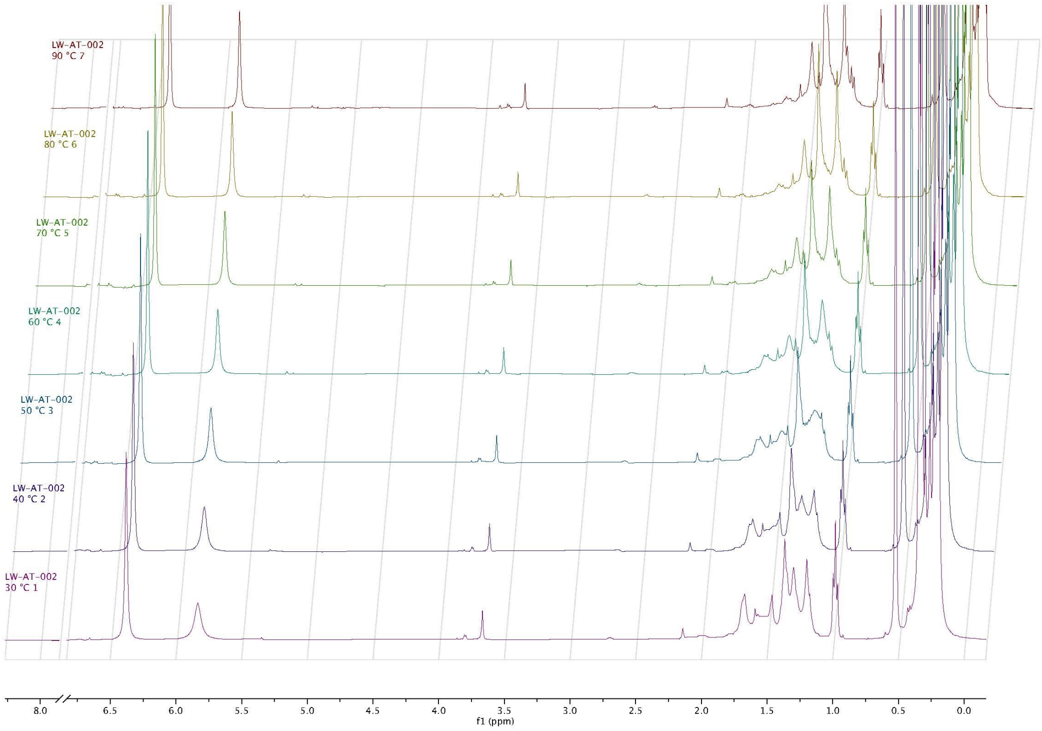

Figure S21: *VT NMR study on the stability of the FRP in the absence of PE, conducted from 30 °C up to 90 °C with 10 °C temperature differences, with TCE as internal standard in 1,1,2,2-tetrachloroethane-d$_2$.*

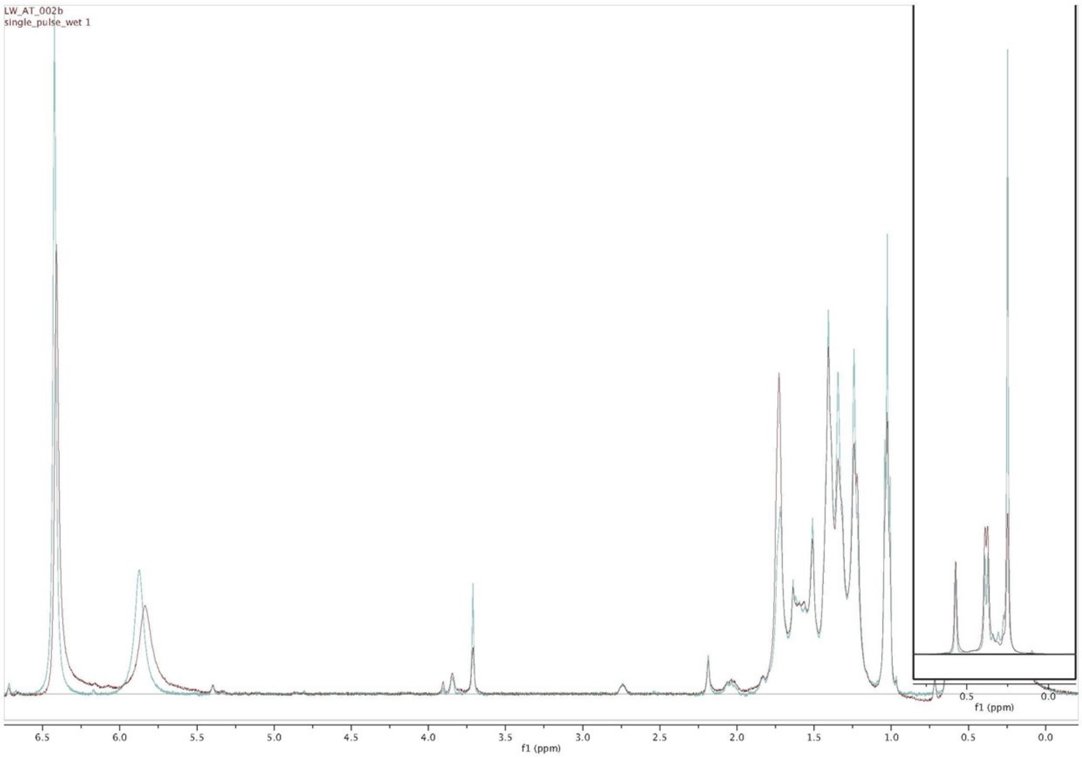

Figure S22: *VT NMR study on the stability of the FRP in the presence of PE during the functionalisation reaction, conducted at 90 °C, with TCE as internal standard in 1,1,2,2-tetrachloroethane-d_2.The blue trace is the mixture prior to heating and the red trace is the reaction mixture after the functionalisation reaction.*

5. DSC and TGA Data

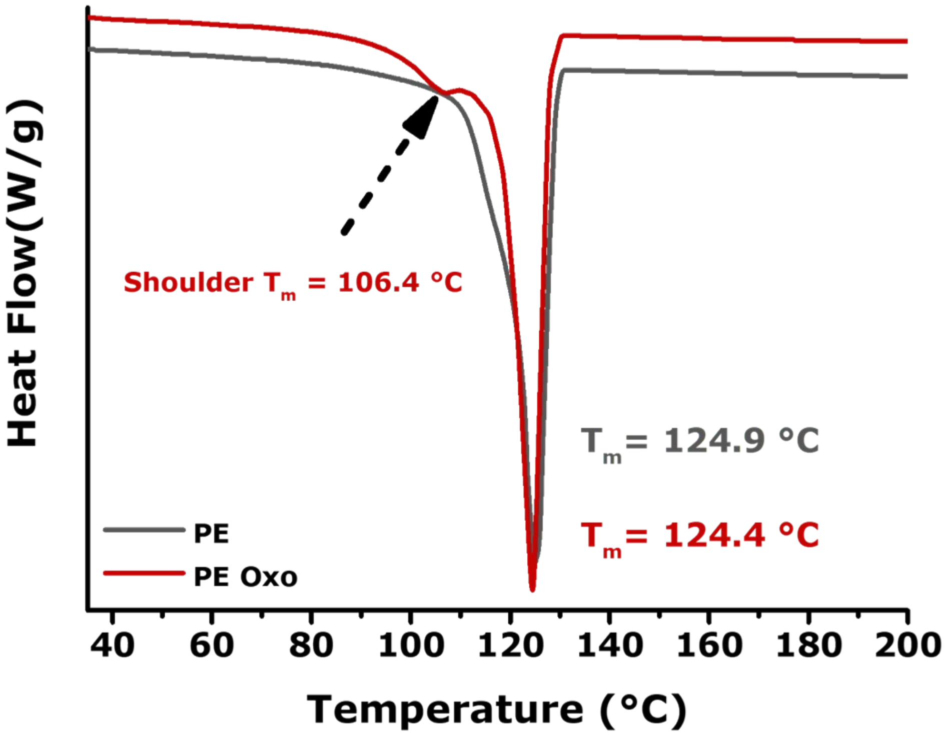

Figure S23: *Differential Scanning Calorimetry (DSC) traces of the reversing heat flow of unfunctionalised polyethylene (grey) and the oxyfunctionalised polyethylene (red).*

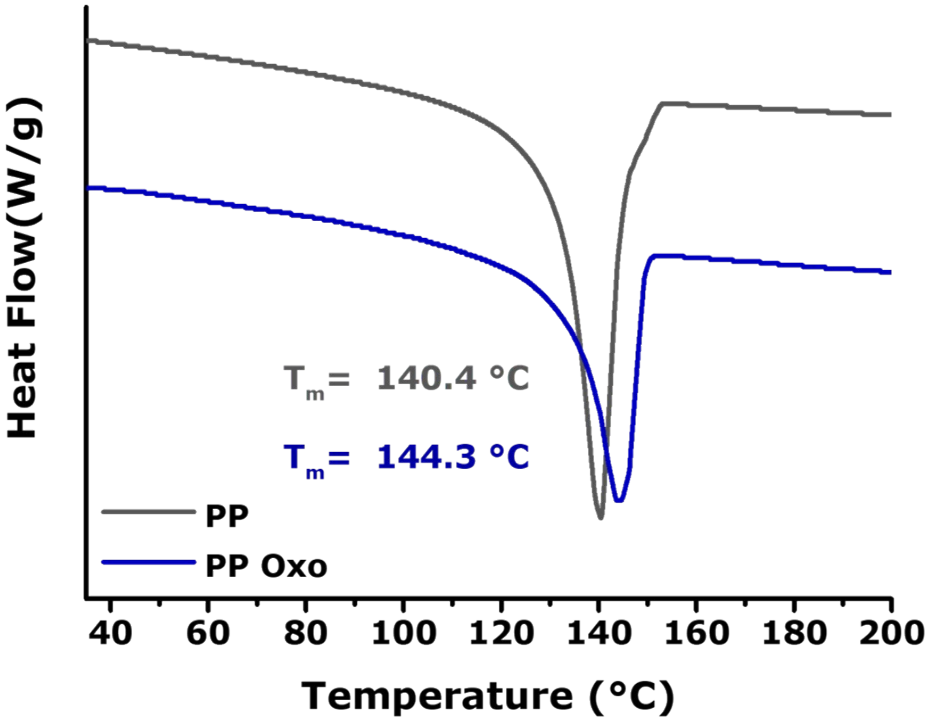

Figure S24: *Differential Scanning Calorimetry (DSC) traces of the reversing heat flow of unfunctionalised polypropylene (grey) and the oxyfunctionalised polypropylene (blue).*

Figure S25: *Thermogravimetric analysis (TGA) traces of unfunctionalised polyethylene (grey) and the oxyfunctionalised polyethylene (red).*

Figure S26: *Thermogravimetric analysis (TGA) traces of unfunctionalised polypropylene (grey) and the oxyfunctionalised polypropylene (blue).*

6. GPC Data

Table S2: *Gel Permeation Chromatography (GPC) data of unfunctionalised polyethylene (PE) and polypropylene (PP) the oxyfunctionalised polymers after the C-H bond oxidation with frustrated radical pairs and mCPBA.*

Polymer	M_w (kDa)	M_n (kDa)	Đ (M_w/M_n)
PE	1.9	1.8	1.06
PE Oxo	2.0	1.8	1.11
PP	6.3	2.7	2.37
PP Oxo	6.2	2.7	2.30

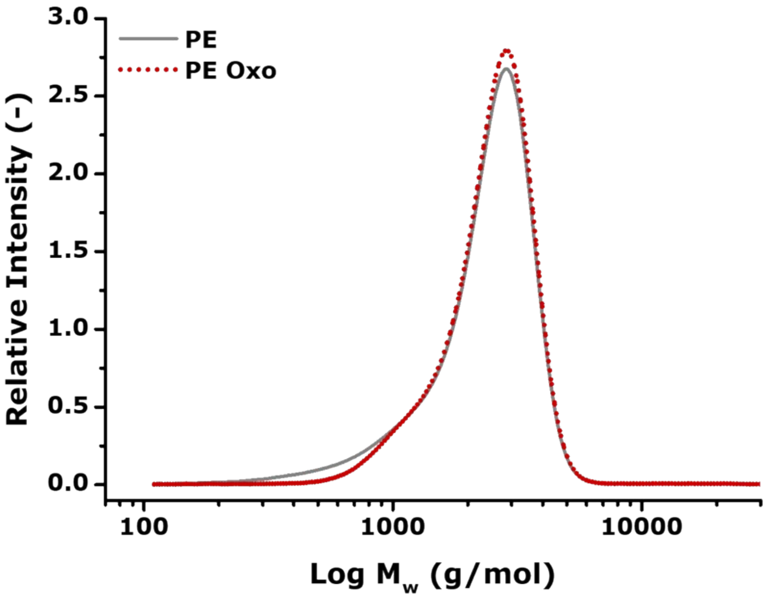

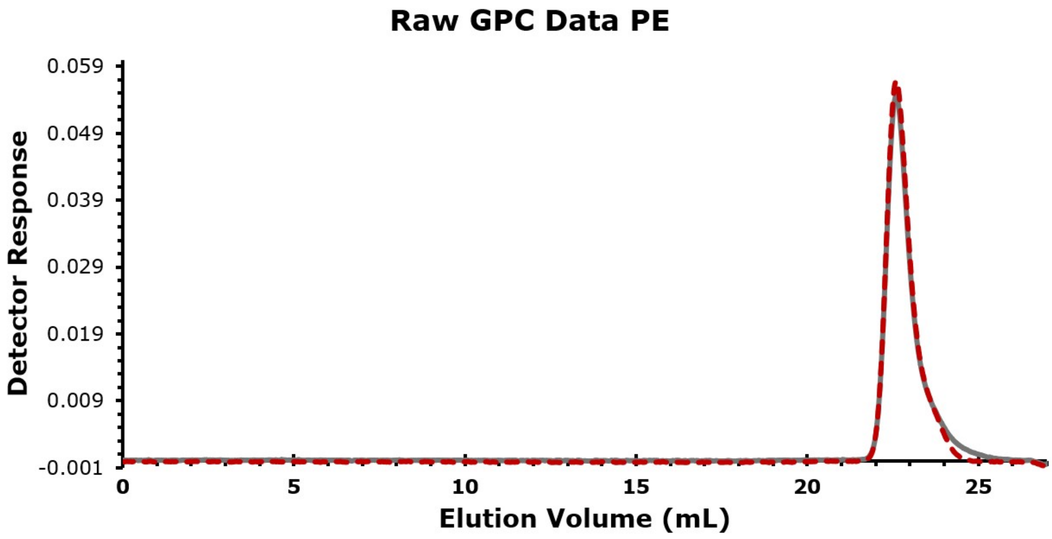

Figure S27: *Gel Permeation Chromatography (GPC) traces of unfunctionalised polyethylene (PE) (grey) and the oxyfunctionalised polyethylene (PE Oxo) (red) (Top) and Raw GPC chromatograms (Bottom).*

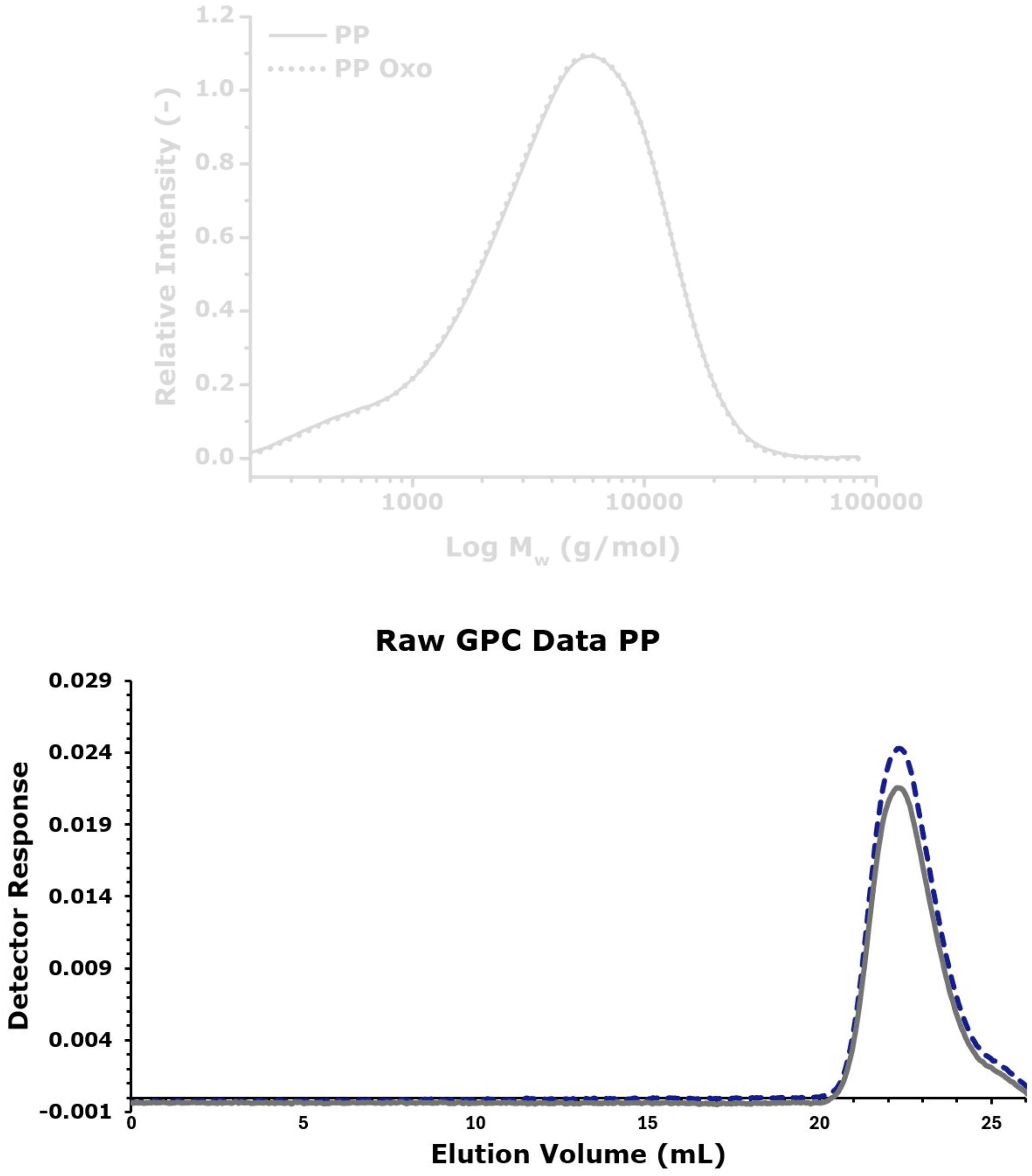

Figure S28: *Gel Permeation Chromatography (GPC) traces of unfunctionalised polypropylene (PP) (grey) and the oxyfunctionalised polypropylene (PP Oxo) (blue) (Top) and Raw GPC chromatograms (Bottom).*

References

(1) Otten, M.; Klein Gebbink, I.; Schara, P. J.; Tomović, Ž.; Lutz, M.; Bruijnincx, P. C. A.; Thevenon, A. Post-Polymerization Modification of Polyethylene through Photochemical Oximation and Consecutive Ketonization. *J. Am. Chem. Soc.* **2025**, *147* (26), 22827–22838.

(2) Borodkin, G. I.; Elanov, I. R.; Shakirov, M. M.; Shubin, V. G. Reaction of Nitrosonium Tetrafluoroborate with Nitroxyl Radicals. *Russ. J. Org. Chem.* **2003**, *39* (8), 1144–1150.

(3) Mercadante, M. A.; Kelly, C. B.; Bobbitt, J. M.; Tilley, L. J.; Leadbeater, N. E. Synthesis of 4-Acetamido-2,2,6,6-Tetramethylpiperidine-1-Oxoammonium Tetrafluoroborate and 4-Acetamido-(2,2,6,6-Tetramethyl-Piperidin-1-yl)Oxyl and Their Use in Oxidative Reactions. *Nat. Protoc.* **2013**, *8* (4), 666–676.

(4) Kim, M. J.; Mun, J.; Kim, J. Oxoammonium Salt-Mediated Oxidative Nitriles Synthesis from Aldehydes with Ammonium Acetate. *Tetrahedron Lett.* **2017**, *58* (50), 4695–4698.

(5) Lu, Z.; Ju, M.; Wang, Y.; Meinhardt, J. M.; Martinez Alvarado, J. I.; Villemure, E.; Terrett, J. A.; Lin, S. Regioselective Aliphatic C–H Functionalization Using Frustrated Radical Pairs. *Nature* **2023**, *619* (7970), 514–520.

(6) Kortman, G. D.; Orr, M. J.; Hull, K. L. Synthesis and Reactivity of Dioxazirconacyclohexenes: Development of a Zirconium-Oxo-Mediated Alkyne-Aldehyde Coupling Reaction. *Organometallics* **2015**, *34* (6), 1013–1016.

(7) Tilley, T. D.; Andersen, R. A. Pentamethylcyclopentadienyl Derivatives of the Trivalent Lanthanide Elements Neodymium, Samarium, and Ytterbium. *Inorg. Chem.* **1981**, *20* (10), 3267–3270.
